# Effects of local climate on loggerhead hatchling production in Brazil: Implications from climate change

**DOI:** 10.1038/s41598-019-45366-x

**Published:** 2019-06-20

**Authors:** Natalie Montero, Pilar Santidrian Tomillo, Vincent S. Saba, Maria A. G. dei Marcovaldi, Milagros López-Mendilaharsu, Alexsandro S. Santos, Mariana M. P. B. Fuentes

**Affiliations:** 1 Department of Earth, Ocean, and Atmospheric Science, Florida State University, Tallahassee, USA; 2 The Leatherback Trust, Goldring-Gund Marine Biology Station, Playa Grande, Costa Rica; 3 National Oceanic and Atmospheric Administration, National Marine Fisheries Service, Northeast Fisheries Science Center, Geophysical Fluid Dynamics Laboratory, Princeton University, 201 Forrestal Road, Princeton, New Jersey USA; 4 Fundação Pró-Tamar, Rubens Guelli, 134, sala 307, Salvador, Bahia Brazil

**Keywords:** Conservation biology, Environmental impact, Marine biology

## Abstract

Sea turtle eggs are heavily influenced by the environment in which they incubate, including effects on hatching success and hatchling viability (hatchling production). It is crucial to understand how the hatchling production of sea turtles is influenced by local climate and how potential changes in climate may impact future hatchling production. Generalized Additive Models were used to determine the relationship of six climatic variables at different temporal scales on loggerhead turtle (*Caretta caretta*) hatchling production at seventeen nesting beaches in Bahia, Espirito Santo, and Rio de Janeiro, Brazil. Using extreme and conservative climate change scenarios throughout the 21^st^ century, potential impacts on future hatching success (the number of hatched eggs in a nest) were predicted using the climatic variable(s) that best described hatchling production at each nesting beach. Air temperature and precipitation were found to be the main drivers of hatchling production throughout Brazil. CMIP5 climate projections are for a warming of air temperature at all sites throughout the 21^st^ century, while projections for precipitation vary regionally. The more tropical nesting beaches in Brazil, such as those in Bahia, are projected to experience declines in hatchling production, while the more temperate nesting beaches, such as those in Rio de Janeiro, are projected to experience increases in hatchling production by the end of the 21^st^ century.

## Introduction

Oviparous reptiles, such as sea turtles, are heavily influenced by environmental temperature, since it can influence their life history, physiology, and behavioral traits, particularly during egg incubation^1–3^. Sex in sea turtles is determined by incubation temperature of the nest, with warmer temperatures producing more females than males^4–6^. Temperatures above the threshold also reduce hatching success, the number of hatched eggs in a nest^5–7^. Considering the influence of the environment on sea turtles and projected increases in temperatures, there is concern over potential impacts on their populations, such as feminization of populations and reduced population stability^8–10^. This concern has prompted an increase in the number of studies exploring climate change impacts on sea turtles (for reviews see^2,11,12^), with most focusing on how predicted changes in temperature will affect the sex ratio of hatchlings^13–15^. However, perhaps of greater threat, and concern, to sea turtle populations is the impact that changes in local climate may have on hatchling production (hatching success and emergence rate), which can impact population growth and stability^7,14,16,17^.

Air temperature, precipitation, and humidity are all known to influence hatchling production, and changes are likely to affect hatching success and emergence rate^4,16,18^. Successful incubation of sea turtle eggs occurs within a narrow thermal range of typically 25 °C to 35 °C, with thermal tolerances varying between species and populations^19–21^. Nevertheless, nest temperatures may exceed 35 °C by several degrees in some populations (usually just prior to hatchling emergence) and eggs still successfully hatch^20^. Despite these varying degrees of tolerance to environmental temperatures, hatchlings incubating in higher temperature nests may exhibit lower hatching success, emergence rate, and smaller size, with higher rates of morphological abnormalities and/or with reduced locomotor performance^22–24^. Indeed, recent work predicts local declines in some populations of leatherback turtles, *Dermochelys coriacea*, (Sandy Point, St. Croix, US Virgin Islands), while other populations are predicted to thrive as a result of drier and warmer conditions due to temperate climate at their nesting beaches (Maputaland, South Africa)^16^.

Considering global projections for changes in climate^25^ and potential implications to hatchling production, it is crucial to understand how sea turtle hatching success and emergence is influenced by different climatic variables at local scales and to forecast potential impacts on sea turtles as climate change progresses. Most studies to date that have explored this have focused primarily on the influence of air temperature and precipitation on hatchling production. We expand on existing work and explore how six climate variables (air and sea surface temperature, rainfall, humidity, solar radiation, and wind speed) influence loggerhead, *Caretta caretta*, hatchling production from the Southwest Atlantic Loggerhead Regional Management Unit^26^ on the coast of Brazil. Regional Management Units assist with grouping sea turtle populations geographically and prioritizing research and conservation^26^. We then identified nesting sites in a large population that are most susceptible to climate change and projected potential effects on hatchling success under extreme (CMIP5 RCP 8.5, higher concentrations of greenhouse gas emissions causing higher increases in global temperatures) and conservative (CMIP5 RCP 4.5, lower concentrations in emission causing more mild increases in global temperatures) scenarios of climate change.

## Methods

### Study Species and Location

**Figure 1:**
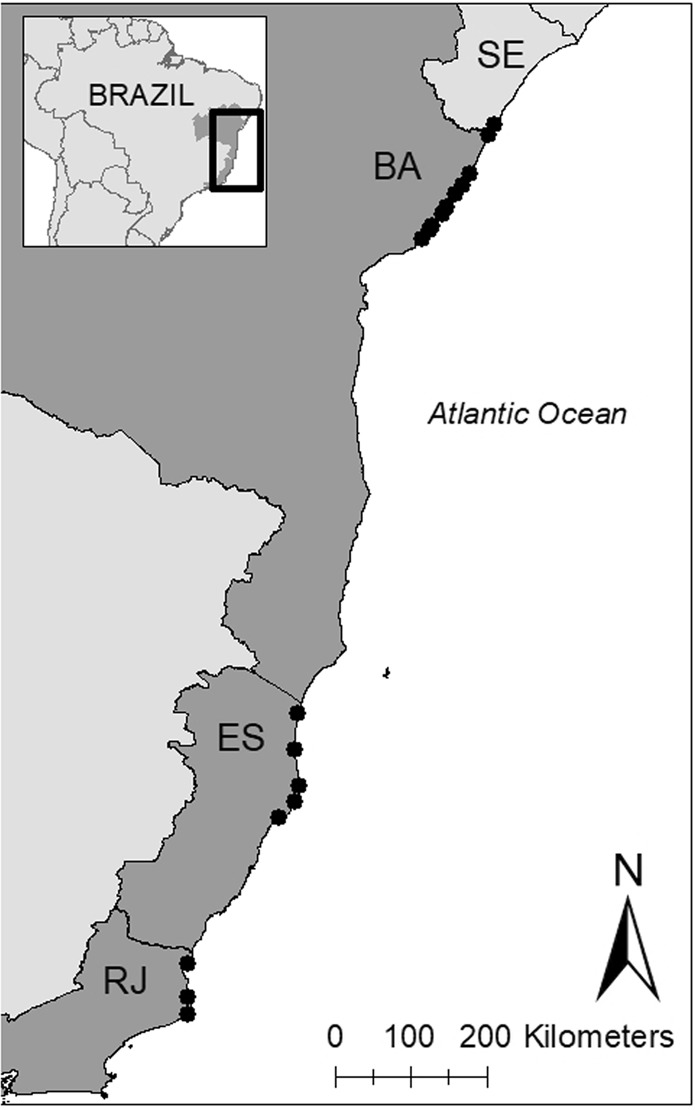
Nesting beaches for loggerhead turtles in Brazil. From north to south the states are Segipe (SE), Bahia (BA), Espírito Santo (ES), and Rio de Janiero (RJ), but SE was not included in the study. Nesting beaches included, from north to south, are: Mangue Seco, Dunas, Siribinha, Baixios, Subauma, Costa do Sauipe, Praia do Forte, Itacimirim, Berta, and Santa Maria in BA; Itaunas, Guriri, Pontal do Ipiranga, Povoacao, and Comboios in ES, and Ilha de Convivencia, and Maria Rosa in RJ.

This study focused on the loggerhead, *Caretta caretta*, population that nests along the Brazilian coast. There are two genetically distinct loggerhead populations in Brazil; the northern stock that nests in Bahia (BA) and Sergipe (SE) and the southern stock that nests in Espírito Santo (ES) and Rio de Janeiro (RJ)^27,28^ (Fig. 1). We used nesting data from seventeen nesting beaches: in Bahia: Mangue Seco, Dunas, Siribinha, Baixios, Subauma, Costa do Sauipe, Praia do Forte, Itacimirim, Berta, Santa Maria; in Espirito Santo: Itaunas, Guriri, Pontal do Ipiranga, Povoacao, and Comboios; in Rio de Janeiro: Ilha de Convivencia and Maria Rosa (Fig. 1). The majority of loggerhead nesting in Brazil (>75%) occurs on the northern coasts of Bahia and Espirito Santo^29^.

### Nest data

Nesting data was obtained from Projeto TAMAR^29^, which conducts daily patrols to monitor nesting activity at these beaches during the sea turtle nesting season (September to March), including nest location, lay date, hatch date, and hatchling production. Loggerhead nesting data from 2005 to 2014, across seventeen nesting grounds (BA = 10, ES = 5, RJ = 2), was incorporated in the study. A total of 17,428 nests were considered for this study (BA = 11,946, ES = 4,006, RJ = 1,476). Due to logistical and financial constraints, neither Espirito Santo nor Rio de Janeiro had consistent nesting data (Table 1). The 2006–2007 nesting season in Rio de Janeiro had fewer than five reported nests in total, so that season was not included in the analyses. Nests from Sergipe, which represented 1.8% of nests for loggerhead turtles were not included in this study because the necessary climate data was not available for this state. We only considered *in situ* nests that had data for hatching success (percentage of hatched eggs within a clutch) and emergence rate (number of hatchlings that emerged from the nest in relation to the total hatched eggs).

**Table 1:** Local hatchling production and climate variables data available for each state included in our analyses.

Bahia months considered (n = 62)	Espirito Santo months considered (n = 42)	Rio de Janeiro months considered (n = 31)
September 2005 - February 2006; September 2006 – March 2007; September 2007 – March 2008; September 2008 – March 2009; September 2009 – March 2010; September 2010 – March 2011; September 2011 – March 2012; September 2012 – March 2013; September 2013 – March 2014	September 2005 – January 2006; October 2006 – January 2007; October 2007 – January 2008; October 2008 – January 2009; October 2009 – January 2010; September 2010 – January 2011; October 2011 – March 2012; September 2012 – January 2013; October 2013 – January 2014	November 2005 – January 2006; October 2007 – January 2008; October 2008 – January 2009; October 2009 – January 2010; October 2010 – February 2011; October 2011 – January 2012; October 2012 – January 2013; October 2013 – January 2014

The typical nesting season in BA is September – March, but, the majority of nesting in ES and RJ takes place between October – January. We included months outside these ranges if more than 1% of nesting occurred in those months. No reliable data exists for RJ for the 2006/2007 nesting season.

### Climate data

Local climate data for 2005 to 2014 was obtained for each of the states considered here. Daily air and sea surface temperatures (°C), as well as precipitation (mm/day) were obtained from the National Oceanic and Atmospheric Administration (NOAA, http://www.ncdc.noaa.gov/cdo-web/datasets) and daily humidity (%), solar radiation (KJ/M^2^), and wind speed (m/s) data was obtained from the Brazilian National Institute of Meteorology (INMET, http://www.inmet.gov.br/portal/). INMET weather stations are in Salvador, Bahia, Vitoria, Espirito Santo, and São Tomé, Rio de Janeiro (Table S4). Air and sea surface temperatures and precipitation were provided in monthly averages for each state. Humidity, solar radiation, precipitation, and wind speed were obtained hourly for each state. To homogenize all the climate data, we averaged the hourly and daily data into monthly averages. Precipitation data from the INMET weather stations were converted to monthly sums to provide accumulated rainfall for each state. Months and seasons where climate data was not available were excluded from any of our analyses (Table 1).

In order to determine potential changes to the incubating environment of loggerhead turtles as climate change progresses and consequent implications to loggerhead hatchling success, we added monthly deltas (the difference between the model projection and the baseline) differences to our historical data for air temperature and precipitation. These deltas gathered from multi – model means for CMIP5 (Coupled Model Intercomparison Project Phase 5) RCP (Representative Concentration Pathways) 8.5 and 4.5 from KNMI Climate Explorer (https://climexp.knmi.nl/start.cgi). CMIP5 RCP 8.5, an extreme scenario, predicts a large increase in air temperatures due to continuous releases of greenhouse gases (GHG) through to the year 2100, creating an extreme scenario^30^. RCP 4.5 presents a more conservative scenario demonstrating stabilized GHG emissions, resulting in a milder increase in air temperatures^30^. We added our monthly deltas for each projection to the average air temperature and accumulated precipitation during each month within the nesting season at each state throughout 2005–2014. This allowed us to explore trends in the predicted climate within each month across projections and years, and consequent implications to hatchling success.

### Analysis

Hatching success and emergence rate data were arcsin transformed to normalize the data. A one-way ANOVA was used to compare hatching success and emergence rate at each state and across nesting beaches within states. The Levene test with center mean was used to test homogeneity of variances in hatching success and emergence rate. The Tukey Honest Significant Difference (TukeyHSD) test was used for hatching success because variances in the data met the assumption of homogeneity (p = 0.101). Tamhane’s test was used for emergence rate because variances in data were not homogenous (p = < 2.2 e-16). ANOVAs on hatching success and the Levene’s test were completed in R version 3.3.2. Version 24 of IBM SPSS Statistics was used to complete the one-way ANOVA and Tamhane’s tests used for emergence rate.

We used Generalized Additive Models (GAM), with the binomial family, to test the non-linear relationships between the six local climate variables and hatching success and emergence rate at different spatial scales. We selected beaches with high nesting densities and spatially representative of each state to explore the influences of climate variables on the reproductive output of loggerheads: Mangue Seco, Praia do Forte, and Santa Maria in Bahia; Itaunas, Povoacao, and Comboios in Espirito Santo, and Maria Rosa in Rio de Janeiro (proportion of nests for each location can be found in Table S2). Due to their representativeness, selected beaches within each state were combined to explore the influence of the climate variables on reproductive output at a state-wide scale. To explore effects at a regional scale, all seven selected beaches were combined. GAMs do not assume a linear relationship, and they estimate the non-parametric function for each predictor parameter^31^. Hatching success and emergence rate data were presented as number of eggs hatched and not hatched, and number of hatchlings that emerged and dead within nests, respectively^31^. Corrected Akaike Information Criterion (AICc) was used to select the best models. The mgcv library in R version 3.0.2 was used for the GAMs.

The predictor variables used in our GAMs were air temperature (temp), average rain (avg_rain), accumulated rain (acc_rain), sea surface temperature (sst), solar radiation (rad), humidity (humid), and wind speed (wind). The predictors month nests were laid (0_climate variable), month nests were laid and one month prior (0_1_climate variable), month nests were laid and two months prior (0_2_climate variable), two months prior to the nests being laid (2_climate variable) and incubation period (inc_climate variable) were explored at various temporal scales. Current research indicates that temperature and precipitation have significant influence on hatchling production^8,13,16^. Consequently, we chose to combine different precipitation predictors with average air temperature during incubation in order to explore their combined effects on hatchling production. Combined models included average air temperature during incubation in combination with one of the following precipitation parameters: average rainfall during the month of nesting (inc_temp + 0_avg_rain), accumulated rainfall in the month of nesting (inc_temp + 0_acc_rain), average rainfall during the nest month and one-month prior (inc_temp + 0_1_avg_rain), accumulated rainfall in the nest month and one-month prior (inc_temp + 0_1_acc_rain), average rainfall in the nest month and two months prior (inc_temp + 0_2_avg_rain), accumulated rainfall in the nest month and two months prior (inc_temp + 0_2_acc_rain), average rainfall in the two months prior to nesting (inc_temp + 2_avg_rain), accumulated rainfall in the two months prior to nesting (inc_temp + 2_acc_rain), average rainfall during incubation (inc_temp + inc_avg_rain) and accumulated rainfall during incubation (inc_temp + inc_acc_rain). A term in the selected models had fewer unique covariate combinations than specific maximum degrees of freedom and we could not run the following analyses: 2_humid (Espirito Santo, Povoacao, Comboios and all combined), 0_2_humid (Espirito Santo, Itaunas, Povoacao and Comboios), 0_1_wind (Espirito Santo, Itaunas, Povoacao and Comboios) and 0_2_wind (Itaunas).

Due to the size of our dataset, we used Generalized Linear Mixed – Models (GLMMs), with the binomial family, to predict the impact of projected climate on hatching success. Specifically, we projected hatching success at nesting beaches within each state that presented significant relationship between climatic variables and hatching success (Praia do Forte (BA), Itaunas (ES), and Maria Rosa (RJ)) using the predictor variables indicated as having low AICc values and being significant in our GAMs (Table 2). Emergence rate was not projected because it can be influenced by many other factors not included in this study (e.g., sand compaction, substrate type, etc.)^32^. The lme4 library in R version 3.4.2 was used for the GLMMs.

**Table 2:** Best fit and most significant models and their relationship for each nesting beach analyzed (Mangue Seco, Praia do Forte, Santa Maria, Comboios, Itaunas, Povoacao, and Maria Rosa) and each state (Bahia, Espirito Santo, and Rio de Janeiro). Bolded variables are the most significant within the model.

Beach	State Hatching Success Model and Effects	Nesting Beach Hatching Success Model	Variable Effects	State Emergence Rate Model and Effects	Nesting Beach Emergence Rate Model	Variable Effects
Mangue Seco, Bahia	**Inc_temp + inc_acc.rain **Relationships unclear	**Inc_temp** + acc.rain_0.2	Low hatching success with high temperatures and low precipitation	**Inc_temp + acc.rain_0.2 **Low emergence rate with low temperatures and low precipitation	**Inc_temp** + inc_acc.rain	Low emergence rate with high temperatures and low precipitation
Praia do Forte, Bahia		**Inc_temp + inc_acc.rain**	Low hatching success with high temperatures and high precipitation		**Inc_temp + acc.rain_0.2**	Low emergence rate with low temperatures and high precipitation
Santa Maria, Bahia		Inc_temp + Inc_acc.rain	Low hatching success with high temperatures and low precipitation		Inc_temp + **inc_acc.rain**	Low emergence rate with high temperatures and low precipitation
Itaunas, Espirito Santo	**Inc_temp** + **acc.rain_2 **Low hatching success with high temperatures and high precipitaion	**Acc.rain_2**	Low hatching success with high precipitation	**Acc.rain_2 **Relationship unclear	**Acc.rain_2**	Relationship unclear
Povoacao, Espirito Santo		Acc.rain_2	Low hatching success with low precipitation		**Acc.rain_0.2**	Low emergence rate with low precipitation
Comboios, Espirito Santo		**Inc_temp** + acc.rain_0.2	Low hatching success with high temperatures and low precipitation		**Acc.rain_0.2**	Low emergence rate with low precipitation
Maria Rosa, Rio de Janeiro	**Inc_temp + acc.rain_0.2** Low hatching success with low temperatures and high precipitation			**Acc.rain_2** Low emergence rate with low precipitation		

## Results

### Hatching success comparisons between and within states

**Figure 2:**
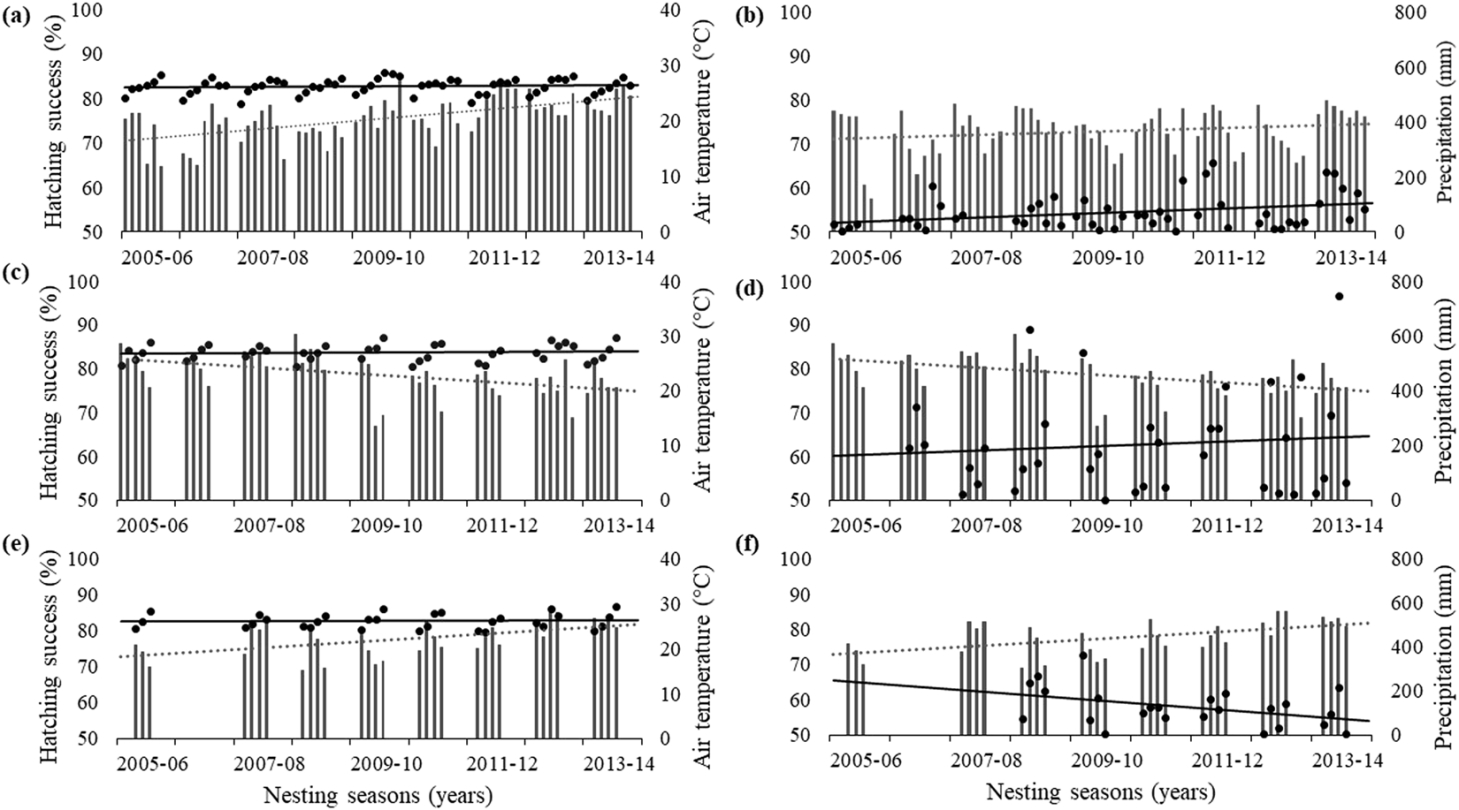
Monthly averages for hatching success (bars) at (**a**,**b**) Bahia, (**c**,**d**) Espirito Santo, and (**e**,**f**) Rio de Janeiro between the 2005/06 to the 2013/14 nesting seasons while the points are monthly averages for (**a**,**c**,**e**) air temperature (°C) and (**b**,**d**,**f**) accumulated rain (mm). Dotted lines are linear trends for hatching success while solid lines are linear climatic trends.

Hatching success varied spatially and temporally across all years (Fig. 2, Table S1). Rio de Janeiro had the highest average hatching success (78.6% ± 19.7), followed by Espirito Santo (78.5% ± 19.3), and Bahia (73.8% ± 19.3). There was a significant difference in hatching success between nesting states across years (ANOVA, DF = 2, F = 142, p < 0.01). The post-hoc test, Tukey HSD, identified a significant difference in hatching success between Bahia and Espirito Santo (p < 0.01) as well as between Bahia and Rio de Janeiro (p < 0.01). No significant difference in hatching success was found between Espirito Santo and Rio de Janeiro (p = 0.998).

In Bahia, a significant difference was found in hatching success among individual nesting beaches (ANOVA, F = 37.58, p < 0.01, DF = 9; Table S2). The nesting beach with highest average hatching success in Bahia was Mangue Seco (81.4% ± 18.4), while the lowest hatching success was found in Santa Maria (73% ± 18.4; Table S1). In Espirito Santo, a significant difference was found in hatching success between individual nesting beaches (ANOVA, F = 4.1, p = 0.003, DF = 4; Table S2). The nesting beach with highest average hatching success was Guriri (82.4% ± 15.9), with the lowest at Comboios (78% ± 19.8; Table S1). A significant difference was found among nesting beaches in Rio de Janeiro (ANOVA, F = 4.3, p < 0.03, DF = 1; Table S2). The average hatching success in Rio de Janeiro varied from 79.2% ± 19.1 in Maria Rosa and 76.6% ± 21.7 at Ilha de Convivencia (Table S1).

### Emergence rate comparisons between and within states

**Figure 3:**
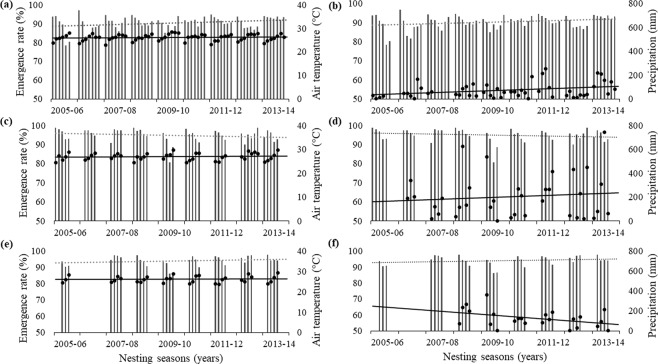
Monthly averages for emergence rate (bars) at (**a**,**b**) Bahia, (**c**,**d**) Espirito Santo, and (**e**,**f**) Rio de Janeiro between the 2005/06 to the 2013/14 nesting seasons while the points are monthly averages for (**a**,**c**,**e**) air temperature (°C) and (**b**,**d**,**f**) accumulated rain (mm). Dotted lines are linear trends for emergence rate while solid lines are linear climatic trends.

Emergence rate varied spatially and temporally at our study sites (Fig. 3). Across all years, Espirito Santo had the highest emergence rate (94.7% ± 8.8), followed by Rio de Janeiro (93.9% ± 8.6) and Bahia (89.7% ± 12.5). There was a significant difference in emergence rate among nesting states (one-way ANOVA, DF = 2, F = 322.9, p < 0.001), with differences between Bahia and Espirito Santo (Tamhane’s test, p < 0.001), Bahia and Rio de Janeiro (Tamhane’s test, p < 0.001), and Espirito Santo and Rio de Janeiro (Tamhane’s test, p = 0.011).

A significant difference in emergence rate was observed across years among nesting grounds within the state of Bahia (ANOVA, F = 50.19, p < 0.001, DF = 9; Table S2). The nesting beach in Bahia with the highest average emergence rate was Mangue Seco (95.6% ± 7.5), with the lowest at Dunas (76.1% ± 9.4; Table S1). Significant differences were found in the emergence rate across years among nesting beaches in Espirito Santo (ANOVA, F = 9.61, p < 0.001, DF = 4; Table S2). The highest average emergence rate in Espirito Santo was at Guriri with 95.8% ± 7.1 while the lowest was at Povoacao is 93.6% ± 9.9 (Table S1). A small significant difference was found in emergence rate across years between nesting beaches at Rio de Janeiro (ANOVA, F = 2.83, p = 0.048, DF = 1; Table S2), with an average emergence rate of 93.2% ± 9.4 at Ilha de Convivencia and 94.1% ± 8.4 at Maria Rosa (Table S1).

### Local climate at nesting grounds during the nesting season

**Figure 4:**
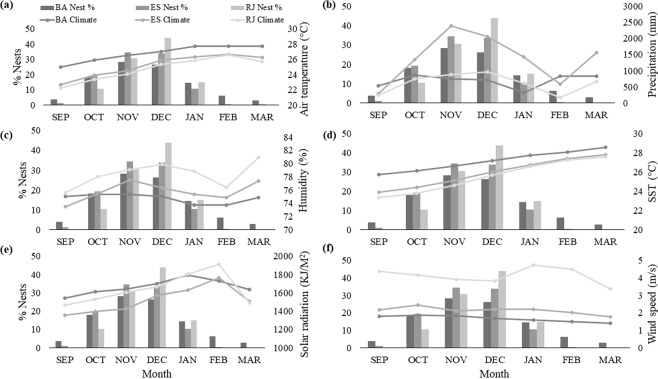
Monthly climatic conditions for each state (line) and proportion of clutches laid within each month (bars) from 2005–2014. Dark gray bars and lines indicate values for Bahia (BA), gray bars and lines indicate values for Espirito Santo (ES), and light gray bars and lines indicate values for Rio de Janeiro (RJ). Climate variables are listed as follows: (**a**) air temperature, (**b**) accumulated precipitation, (**c**) humidity, (**d**) sea surface temperature, (**e**) solar radiation, and (**f**) wind speed.

The nesting season for loggerhead turtles in Brazil takes place during the warmest and wetter months of the year, September – March. During the nesting season, Rio de Janeiro is the most humid state with March being the most humid month, correlating with the lowest nesting activity in the state (Fig. 4b). In contrast, in Espirito Santo and Bahia, November is the most humid month, correlating with a peak in nesting (Fig. 4b). January and February had the highest solar radiation, coinciding with low nesting across all states (Fig. 4c). Rio de Janeiro has the most rainfall with December experiencing a peak in both rainfall and nesting activity (Fig. 4d). In Bahia and Espirito Santo, peak rainfall occurs in the early, October, and late, February, stages of their nesting seasons (Fig. 4d). Like air temperature, months with the coolest, September and October, and warmest, February and March, SST correspond to the lowest nesting across states (Fig. 4a,e). Months with the lowest average wind speed in Bahia and Espirito Santo coincides with the lowest nesting, whereas January and February have the highest average wind speeds in Rio de Janeiro, but the lowest nesting (Fig. 4f).

### Effect of local climatic conditions on hatchling production

Overall, the best GAM for hatching success within the entire region was average air temperature during incubation in combination with accumulated rainfall during incubation (deviance = 8.94%; Fig. S1; Table S3). Both air temperature and precipitation were significant (p < 0.01 for both parameters), but their relationships are unclear (Fig. S1). This same model had the lowest Corrected Akaike Information Criterion (AICc) value across emergence rate models throughout the region (deviance = 14.2%; Fig. S1; Table S3). Average air temperature during incubation had a non-significant negative effect on emergence rate (p = 0.27), while accumulated rainfall during incubation had a highly significant and negative effect on emergence rate (p < 0.01; Table 2; Fig. S1; Table S3).

#### Effect of local climatic conditions on hatching success

**Figure 5:**
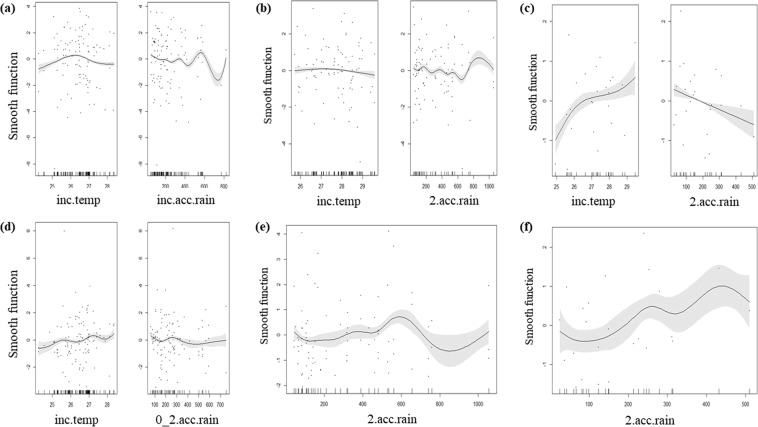
Best fit models descibing (**a**–**c**) hatching success and (**d**–**f**) emergence rate at each state (**a**,**d**) Bahia, (**b**,**e**) Espirito Santo, and (**c**,**f**) Rio de Janeiro) using AICc, deviance, and p-values. At (**a**) Bahia the best model describing hatching success was average air temperature during incubation (inc.temp) and accumulated rainfall during incubation (inc.acc.rain). The best model at (**b**) Espirito Santo describing hatching success was average air temperature during incubation (inc.temp) and accumulated rainfall in the two months prior to nests being laid (2.acc.rain). In (**c**) Rio de Janeiro the best model was describing hatching success average air temperature during incubation (inc.temp) and accumulated rainfall in the two months prior to nests being laid (2.acc.rain). The (**d**) best emergence rate model at Bahia was average air temperature during incubation (inc.temp) and accumulated rainfall during the nest month and two months prior (0.2.acc.rain). The (**e**) best model describing emergence rate at Espirito Santo was accumulated rainfall in the two months prior to nests being laid (2.acc.rain). In (**f**) Rio de Janeiro the best emergence rate model was accumulated rainfall in the two months prior to nests being laid (2.acc.rain).

In Bahia, the best model (lowest AICc) for hatching success was average air temperature during incubation with accumulated rain during incubation (deviance = 26.5%; Fig. 5a; Table S3). Both air temperature and precipitation were significant (p < 0.01 for both parameters), with lower hatching success occurring at warmer and cooler temperatures, while the relationship with precipitation was unclear (Fig. 5a). In Espirito Santo, the best model for hatching success was average air temperature during incubation with accumulated rainfall in the two months prior to nesting (deviance = 28.9%; Fig. 5b; Table S3). Within this model, air temperature during incubation was significant and had a negative effect (p = 0.04), while accumulated rainfall in the two months prior to nesting was highly significant and had a negative effect (p < 0.01; Fig. 5b). In Rio de Janeiro, the best model was air temperature during incubation with accumulated rain in two months prior to the nest month (deviance = 70.7%, p < 0.01 for both parameters; Fig. 5c, Table S4). In this model, air temperature had a positive effect while precipitation had a negative effect (Table 2). Effects of local climatic conditions on hatching success were analyzed for individual beaches within each state (Table 2 and Table S4).

#### Effect of local climatic conditions on emergence rate

The best model for emergence rate within Bahia was average air temperature during incubation with accumulated rainfall in the nest month and two months prior (deviance = 18.9%, p < 0.01 for both parameters; Fig. 5d; Table S4). Both temperature and precipitation had a positive relationship (Table 2). The best model for emergence rate in Espirito Santo was accumulated rain in the two months prior to nesting (deviance = 24.1%, p < 0.01; Fig. 5e; Table S4). There was no clear relationship of precipitation on emergence rate (Fig. 5e; Table 2). In Rio de Janeiro, the best model was accumulated rain in the two months prior to the nest month (deviance = 60.7%, p < 0.01; Fig. 5f; Table S4). There was a positive effect of precipitation on emergence rate (Table 2). Effects of local climatic conditions on emergence rate were analyzed for individual beaches within each state (Table 2 and Table S4).

### Projections

#### Climate

**Figure 6:**
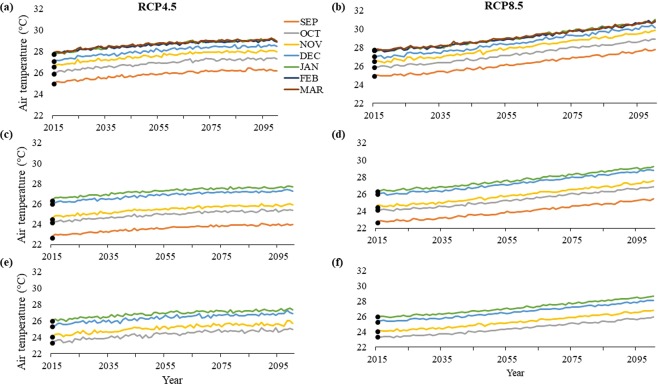
Historic averages (solid points) and projected values of air temperature (°C) for (**a**,**b**) Praia do Forte, Bahia, (**c**,**d**) Itaunas, Espirito Santo, (**e**,**f**) and Maria Rosa, Rio de Janeiro under the extreme (**b**,**d**,**f**) CMIP5 RCP8.5 scenario and the more conservative (**a**,**c**,**e**) CMIP5 RCP4.5 scenario. Espirito Santo and Rio de Janeiro have fewer months shown to reflect their shorter nesting seasons compared to Bahia.

Increases in air temperature is projected across the study sites throughout the 21^st^ century (Fig. 6). By 2100, climate models project air temperatures to increase in Bahia by 1.1–3.3 °C throughout the nesting season, with the highest temperatures in March (Fig. 6a,b). In Espirito Santo, air temperature is projected to increase by 1.3–3.1 °C, with highest temperatures in January (Fig. 6c,d). Rio de Janeiro is projected to experience the lowest increase in temperature of 1.3–2.8 °C, with the highest temperature increase in January (Fig. 6e,f).

**Figure 7:**
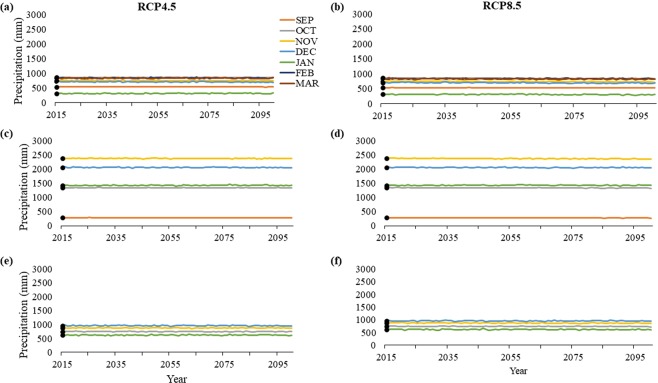
Historic averages (solid points) and projected values of precipitation (mm) for (**a**,**b**) Praia do Forte, Bahia, (**c**,**d**) Itaunas, Espirito Santo, (**e**,**f**) and Maria Rosa, Rio de Janeiro under the extreme (**b**,**d**,**f**) CMIP5 RCP8.5 scenario and the more conservative (**a**,**c**,**e**) CMIP5 RCP4.5 scenario. Espirito Santo and Rio de Janeiro have fewer months shown to reflect their shorter nesting seasons compared to Bahia.

Projections for accumulated precipitation vary throughout the 21^st^ century and across states (Fig. 7). In Bahia, by 2100 under RCP8.5, climate models project precipitation to decrease by 9.1–35.3 mm in September – December and February – March; however, January is projected to experience an increase by 5.7 mm (Fig. 7a). Climate models under RCP4.5 project a decrease in precipitation by 5.8–20.2 mm in September – December. While increases are projected for January – March by 12.1–22.4 mm (Fig. 7b). In Espirito Santo, precipitation is projected to decrease by 5.5–30.2 mm in September – December but increase in January by 2.5–9.2 mm (Fig. 7c,d). In Rio de Janeiro, climate models under RCP8.5 project decreases by 9–26.7 mm in October – January (Fig. 7e), whereas RCP4.5 projects decreases by 3.1–17.3 mm in November – January but increase slightly in October by 0.1 mm (Fig. 7f).

#### Hatching Success projections

**Figure 8:**
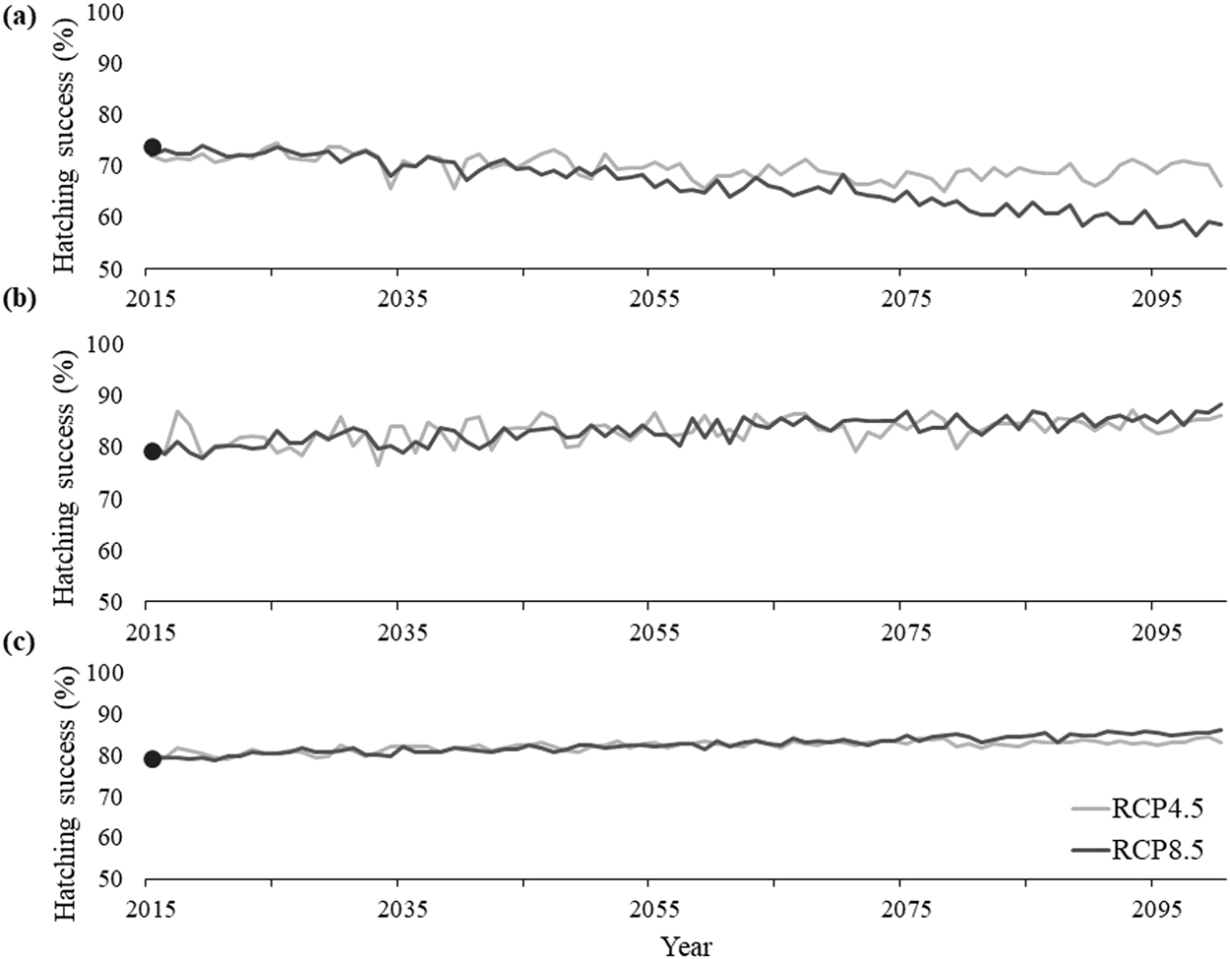
Historic average (solid point) and projected hatching success at (**a**) Praia do Forte, Bahia, (**b**) Itaunas, Espirito Santo, and (**c**) Maria Rosa, Rio de Janeiro under the extreme (light blue) CMIP5 RCP8.5 scenario and the more conservative (gray) CMIP5 RCP4.5 scenario.

By 2100, hatching success is projected to decrease in Bahia from an average of 69.6% to 58.5% under RCP8.5 and decrease to 66.1% under RCP4.5 by 2100 (Fig. 8a). At Espirito Santo, hatchling success is projected to increase from 79.3% to 88.2% under RCP8.5 and increase to 86% under RCP4.5 (Fig. 8b). Hatching success is projected to increase in Rio de Janeiro from an average of 79.2% to 83.1% under RCP8.5 and increase to 86.2% under RCP4.5 (Fig. 8c).

## Discussion

Accumulated precipitation alone or in combination with air temperature during incubation were the main climatic drivers of loggerhead hatchling production at Brazilian nesting beaches, with the effects of each climatic variable varying among states and nesting beaches. At beaches where precipitation was a significant driver of hatching success, there was a negative effect of precipitation, which could be a result of soil saturation or a rise of the water table level displacing air in between sand particles suffocating embryos resulting in clutch failure^16,33,34^. High levels of precipitation at loggerhead nesting beaches in Brazil can exacerbate the negative effects beyond the moisture threshold for this population since these areas already experience high levels of rain, particularly during the nesting season. Precipitation may have a positive effect on hatchling production in areas that are generally dry during the nesting season, as the lack of rain in these areas reduces developmental success and hatchling emergence^16^. Similarly, at our study sites, precipitation showed a positive relationship with hatchling emergence. This might reflect the fact that drier sands typically makes it harder for hatchlings to emerge from nests due to cave-ins^35^. Ultimately, the effect of precipitation on nesting beaches and sea turtle reproductive output will depend on the environmental conditions of each beach and how close incubating eggs are to threshold levels of moisture and temperature.

Temperature had a larger influence on hatchling production at nesting beaches in Bahia, which are closer to the equator and thus warmer than the other beaches studied here. Generally, we found high and low air temperatures having a negative effect on hatching production. This is not surprising because sea turtle eggs successfully incubate within a narrow thermal range (25 °C and 35 °C - with thermal tolerances varying between species and populations) and temperatures that are too high or too low reduce hatchling success and emergence rates^7,19–21,36^. Thus, nesting beaches that have temperatures close to the thermal threshold for successful incubation will likely experience a significant effect of temperature on hatchling production. This was the case at our study sites, where hatching success was negatively affected by high temperatures at warmer beaches (Bahia) and negatively affected by lower temperatures at cooler beaches (Rio de Janeiro).

The effects of temperature on hatchling production is relatively well understood^37–39^, with recent advancements in our understanding of the effects of precipitation^40–42^. Other less studied climatic variables might also be affecting the reproductive output of sea turtles at nesting grounds. Here, we also explored how solar radiation, wind speeds, humidity, and sea surface temperature may influence hatchling production. Although our study suggests temperature and precipitation to be most significant, another variable, solar radiation, was indicated as a good predictor with a negative effect on hatchling production at multiple nesting beaches. Solar radiation is known to have a negative effect on sea turtle hatchlings due to warmer nest temperatures being associated with low hatching success, smaller carapace sizes in hatchlings, and decreased performance^43^. Thus, there is the need to further explore the effects of other climatic variables at various temporal scales and their combined effects on hatchling production.

As climate change progresses, warmer nesting beaches (Bahia) will experience a reduction in hatching success, whereas the cooler beaches (Espirito Santo and Rio de Janeiro) at our study sites will experience an increase in hatching success. Bahia’s proximity to the equator, and warmer temperatures, meant that beaches in these areas were more affected by projected increases in temperature, whereas Espirito Santo and Rio the Janeiro were more influenced by precipitation, since the temperature at these locations are not close to thermal thresholds for sea turtle incubation. As the effect of precipitation varied within beaches in ES, it is likely that hatchling production will be affected differently as climate change progresses across nesting beaches in the region. Even though, hatchling success was projected to increase in ES and RJ by 2100, it is likely that as climate change progresses and temperatures and precipitation levels approach negative thresholds, hatchling production at these locations will start to decrease.

Similar to our study other loggerhead nesting beaches in the Mediterranean are predicted to experience increases in hatching success by 10%+ by 2080, but continued warming in the future may result in declines^44^. In the short-term, nesting beaches at more temperate regions, e.g., Rio the Janeiro, may likely be less impacted by changes in our climate. Indeed, previous research suggests that ectothermic populations in temperate regions may be more resilient to climate change due to having more seasonal variability than tropical regions^45–47^. Months that are presently too cold for suitable incubation, in places like Rio de Janeiro, may become more suitable as climate change progresses. Similarly, at warmer locations the suitability of incubation conditions may shift to cooler months. These changes can result in shifts in nesting phenology, which has already been observed in response to warmer temperatures causing adult loggerhead females to nest earlier in tropical South Florida^48^. Temporal shifts in nesting in response to warmer temperatures may also result in shorter nesting seasons, which has been observed in loggerheads nesting in Florida^49^. Short nesting seasons are typical of temperate regions, as can be seen in Rio de Janeiro. However, with projected changes in climate, nesting seasons in Rio de Janeiro may lengthen while nesting seasons in Bahia may shift and shorten. Since hatchlings emerging from beaches in the north of Brazil experience different current regimes offshore, depending on the time of year they hatch,^50–53^ it is likely that changes in nesting/hatchling season will affect hatchling dispersal patterns in the region. Any shifts in nesting season and dispersal patterns will require management and conservation initiatives to respond accordingly^54^.

To properly understand the impacts of climate change on sea turtles and their response we also need to consider other potential effects from climate change that can occur concurrently with changes in the climatic variables studied here. Increases in beach erosion and reduced nesting habitat availability due to sea level rise, increase in storm events and severity, and coastal development may negatively affect the reproductive output and viability of nesting beaches that were identified as most resilient to changes in climatic variables^55–59^. The synergetic effects of various climatic variables also need to be considered, as well as how the local climate influences and interacts with the nest microclimate. Indeed, recent studies have started to explore the interactions between the hydric and thermal environment of the nesting environment and influences on embryonic development and phenotype^13,60,61^. The heterogeneous nature of our results and that of previous research indicates that the drivers of hatchling production need to be explored at a nesting beach level, as the effects of climatic variables varied regionally. It is also likely that these relationships will vary among species, since thermal thresholds for sea turtle species also vary^20,21^ and ultimately, influence how various species and populations are affected by climatic changes.

## Supplementary information

Supplementary information
