# Supplementary material for: Effects of local climate on loggerhead hatchling production in Brazil: Implications from climate change
Source: Sci Rep. 2019 Jun 20;9:8861. doi: 10.1038/s41598-019-45366-x (PMC6586835; doi:10.1038/s41598-019-45366-x)
Supplement: Supplementary file 1 — Supplementary information [file 41598_2019_45366_MOESM1_ESM.pdf]

Effects of local climate on loggerhead hatchling production in Brazil: Implications from climate change

Natalie Montero[1]; Pilar Santidrian Tomillo[2], Vincent Saba[3], Maria A.G.dei Marcovaldi[4], Milagros López-Mendilaharsu[4], Alexsandro S. Santos[4], Mariana M.P.B. Fuentes[1*]

[1] Department of Earth, Ocean, and Atmospheric Science, Florida State University 117 N Woodward Ave Tallahassee, FL, USA 32306

[2]The Leatherback Trust, Goldring-Gund Marine Biology Station, Playa Grande, Costa Rica

[3]National Oceanic and Atmospheric Administration, National Marine Fisheries Service, Northeast Fisheries Science Center, Geophysical Fluid Dynamics Laboratory, Princeton University, 201 Forrestal Road Princeton, New Jersey, USA

[4]Fundação Pró-Tamar, Rubens Guelli, 134, sala 307, Salvador, Bahia, Brazil

Table S1. Average monthly hatching success and emergence rate for all years considered per beach as well as the number and proportion of total nesting and the standard deviation at each nesting beach.

State	Beach	Number of Nests (Proportion)	Month	Hatching Success (% ± SD)	Emergence Rate (%± SD)
BA	Mangue Seco	418 (3.5%)	September	87.7 ± 11	99.5 ± 0.7
			October	84.3 ± 15.5	97 ± 5
			November	84.1 ± 16	96.8 ± 5.1
			December	81.2 ± 18.7	94.6 ± 8.9
			January	78.4 ± 18.6	92.8 ± 11.8
			February	76.9 ± 20.5	94.7 ± 6
			March	61.4 ± 31.4	92.3 ± 7.2
			**Total**	**81.4 ± 18.4**	**95.6 ± 7.5**
	Dunas	167 (1.4%)	September	81.7 ± 6.7	97.1 ± 3.7
			October	82.7 ± 14.1	95.1 ± 8.2
			November	71.4 ± 23.5	95.2 ± 6.9
			December	74.4 ± 23.6	93.2 ± 11.9
			January	81.4 ± 10.8	95.8 ± 3.3
			February	64.3 ± 20.4	83.7 ± 24.2
			March	NA	NA
			**Total**	**76.2 ± 20.2**	**76.1 ± 9.4**
	Siribinha	453 (3.8%)	September	73.9 ± 18.6	96.2 ± 3.8
			October	75.7 ± 17.6	94.7 ± 7.1
			November	75.2 ± 18.3	92.4 ± 9.1
			December	73.5 ± 20	89.3 ± 13
			January	67.9 ± 20.8	88.4 ± 11.8
			February	57.6 ± 25.8	90.6 ± 9.1
			March	74.7 ± 18.1	92.6 ±7.2
			**Total**	**72.7 ± 19.8**	**91.3 ±10.6**
	Baixios	501 (4.2%)	September	77.6 ± 17.8	91.5 ± 8.1
			October	74.2 ± 18.4	89.7 ± 11.5
			November	76.8 ± 16.1	87.8 ± 12.8
			December	74.2 ± 17.6	87.6 ± 11.8
			January	73.3 ± 18.8	89 ± 11.2
			February	75.6 ± 16.6	89.4 ± 10.3
			March	82.3 ± 8.3	92.3 ± 3.9
			**Total**	**75.3 ±17.4**	**88.7 ± 11.6**
	Subauma	692 (5.8%)	September	78.4 ± 18.4	94.8 ± 6.3
			October	77.7 ± 19.6	90.8 ± 15
			November	74.7 ± 20.6	91.4 ± 11.8
			December	73.9 ± 19.7	90.5 ± 12.7

			January	70.6 ± 22.4	91.6 ± 11.2
			February	69.5 ± 21.9	93.5 ± 6.1
			March	65.4 ± 29.9	96.9 ± 5.3
			**Total**	**74.2 ± 20.6**	**91.3 ± 12.2**
	Costa do Sauipe	750 (6.3%)	September	74.9 ± 11	96 ± 0.7
			October	75.3 ± 15.5	94.6 ± 5
			November	76.5 ± 16	92 ± 5.1
			December	74.9 ± 18.7	91.7 ± 8.9
			January	74.7 ± 18.6	90.9 ± 11.8
			February	72.6 ± 20.5	91.2 ± 6
			March	70.1 ± 30.5	95 ± 7.2
			**Total**	**75 ± 20.9**	**92.3 ± 11.6**
	Praia do Forte	2,459 (20.6%)	September	67 ± 21.6	93.8 ± 8.9
			October	72.8 ± 16.4	92.3 ± 10.9
			November	72 ± 17.9	91 ± 12
			December	67 ± 20.2	91 ± 11.1
			January	66.3 ± 22.2	90.1 ± 12.2
			February	62.9 ± 23.9	88.6 ± 15.1
			March	65.8 ± 24.7	91 ± 13.8
			**Total**	**69.6 ± 20.2**	**91 ± 12**
	Itacimirim	1,098 (9.2%)	September	81.7 ± 17.2	94.1 ± 8.9
			October	77.1 ± 19.9	93.5 ± 10.8
			November	77.1 ± 18.4	91.3 ± 11.9
			December	76.5 ± 19.8	90.6 ± 12.7
			January	69.4 ± 22.2	84.9 ± 17.8
			February	66.2 ± 24.3	85.9 ± 13.5
			March	81.5 ± 10.6	87.9 ± 11.7
			**Total**	**76.1 ± 19.8**	**90.9 ± 12.8**
	Berta	1,145 (9.6%)	September	82 ± 11.3	94.7 ± 6.4
			October	80.4 ± 16.7	93.8 ± 7.7
			November	80.7 ± 14.9	91 ± 9.9
			December	81.5 ± 15.4	91.3 ± 9.7
			January	75.9 ± 17.2	88.1 ± 11.5
			February	75.2 ± 16.6	89.1 ± 7.8
			March	74.6 ± 12.6	91.2 ± 7.4
			**Total**	**79.9 ± 15.8**	**91 ± 9.8**
	Santa Maria	4,263 (35.7%)	September	74 ± 17.9	91.8 ± 9.3
			October	75.4 ± 16.6	89.7 ± 11.5
			November	73.4 ± 18.2	86.1 ± 14.3
			December	72.4 ± 18.9	85.4 ± 14.4
			January	68.7 ± 20.6	84.3 ± 15.7
			February	72.7 ± 16.5	87.5 ± 12.3
			March	76.7 ± 17.3	91.4 ± 9
			**Total**	**73 ± 18.4**	**86.9 ± 13.8**
ES	Itaunas	200 (5%)	September	NA	NA

			October	81 ± 18.6	97.1 ± 4.5
			November	81 ± 16.6	94.5 ± 10.5
			December	75.8 ± 20.4	95.6 ± 5.6
			January	85.7 ± 13.3	95.1 ± 7.9
			February	NA	NA
			March	NA	NA
			**Total**	**79.3 ± 18.9**	**95.3 ± 8.3**
	Guriri	146 (3.7%)	September	NA	NA
			October	81.1 ± 15.3	97 ± 5.6
			November	84.3 ± 15	96.9 ± 4.2
			December	79.7 ± 18.8	92.3 ± 10.9
			January	92.1 ± 3.9	98.2 ± 2.4
			February	NA	NA
			March	NA	NA
			**Total**	**82.4 ± 15.9**	**95.8 ± 7.1**
	Pontal do Ipiranga	216 (5.4%)	September	74 ± 29.7	96.5 ± 5
			October	81.4 ± 17	96 ± 5.5
			November	83.2 ± 13.1	95.6 ± 4.8
			December	82 ± 12.2	94.4 ± 7.3
			January	82.9 ± 12.9	93.3 ± 9.4
			February	87 ± 1.4	95.4 ± 0.8
			March	NA	NA
			**Total**	**82.1 ± 14.1**	**94.9 ± 6.6**
	Povoacao	1,534 (38.3%)	September	85.1 ± 18	97.9 ± 3.6
			October	81.2 ± 17.6	96.8 ± 5.1
			November	80.3 ± 18.2	94.4 ± 8.3
			December	76.2 ± 20.4	91.3 ± 11.9
			January	72.1 ± 22.7	91.1 ± 12.8
			February	83 ± 23.2	97.4 ± 4.9
			March	68.8 ± 30.4	98.7 ± 2.1
			**Total**	**78.3 ± 19.7**	**93.6 ± 9.9**
	Comboios	1,910 (47.7%)	September	75.3 ± 23.2	95 ± 10.2
			October	79.8 ± 19.6	97 ± 4.3
			November	79.2 ± 18.6	96.4 ± 5.9
			December	77.2 ± 19.8	93.9 ± 10.3
			January	73.9 ± 22.4	93.7 ± 9.8
			February	73.1 ± 24.6	94.7 ± 11.9
			March	NA	NA
			**Total**	**78 ± 19.8**	**95.3 ± 8.1**
RJ	Ilha do Convivencia	306 (20.7%)	September	NA	NA
			October	71.7 ± 25.2	95 ± 4.9
			November	76.5 ± 19	92.3 ± 11.6
			December	80.2 ± 20.6	94 ± 8.3
			January	71.2 ± 26	91 ± 9.4
			February	NA	NA

			March	NA	NA
			**Total**	**76.6 ± 21.7**	**93.2 ± 9.4**
	Maria Rosa	1,170 (79.3%)	September	NA	NA
			October	79.5 ± 17.9	96.6 ± 4
			November	80.4 ± 18.2	95.3 ± 5.7
			December	79 ± 19.7	93.5 ± 8.8
			January	77.4 ± 20	91.9 ± 11.7
			February	NA	NA
			March	NA	NA
			**Total**	**79.2 ± 19.1**	**94.1 ± 8.4**

Table S2. Statistical differences, using Tukey Honest Significant Difference and Tamhane's Test, in hatching success and emergence rate across nesting grounds within each state. Statistical significance comparisons are indicated in bold.

State	Nesting grounds	Hatching Success	Emergence Rate
BA	Berta – Baixios	**0.000**	**0.001**
	Costa do Sauipe – Baixios	1.000	**0.000**
	Dunas – Baixios	0.999	**0.000**
	Itacimirim - Baixios	0.969	**0.000**
	Mangue Seco – Baixios	**0.000**	**0.000**
	Praia do Forte – Baxios	**0.000**	**0.000**
	Santa Maria – Baixios	0.218	0.817
	Siribinha – Baixios	0.735	0.927
	Subauma – Baxios	0.999	**0.000**
	Costa do Sauipe – Berta	**0.000**	**0.000**
	Dunas – Berta	0.336	**0.000**
	Itacimirim – Berta	**0.000**	0.295
	Mangue Seco – Berta	0.530	**0.000**
	Praia do Forte – Berta	**0.000**	0.396
	Santa Maria – Berta	**0.000**	**0.000**
	Siribinha – Berta	**0.000**	**0.000**
	Subauma – Berta	**0.000**	0.159
	Dunas – Costa do Sauipe	0.999	0.251
	Itacimirim – Costa do Sauipe	0.946	0.248
	Mangue Seco – Costa do Sauipe	**0.000**	**0.000**
	Praia do Forte – Costa do Sauipe	**0.000**	**0.006**
	Santa Maria – Costa do Sauipe	**0.047**	**0.000**
	Siribinha – Costa do Sauipe	0.589	0.422
	Subauma – Costa do Sauipe	0.999	0.881
	Itacimirim – Dunas	1.000	**0.000**
	Mangue Seco – Dunas	**0.018**	0.964

	Praia do Forte – Dunas	**0.000**	**0.000**
	Santa Maria – Dunas	0.413	**0.000**
	Siribinha – Dunas	0.666	**0.001**
	Subauma -Dunas	0.989	**0.003**
	Mangue Seco – Itacimirim	**0.000**	**0.000**
	Praia do Forte – Itacimirim	**0.000**	1.000
	Santa Maria – Itacimirim	**0.000**	**0.000**
	Siribinha – Itacimirim	**0.036**	1.000
	Subauma – Itacimirim	0.560	1.000
	Praia do Forte – Mangue Seco	**0.000**	**0.000**
	Santa Maria – Mangue Seco	**0.000**	**0.000**
	Siribinha – Mangue Seco	**0.000**	**0.000**
	Subauma – Mangue Seco	**0.000**	**0.000**
	Santa Maria – Praia do Forte	**0.000**	**0.000**
	Siribinha – Praia do Forte	**0.010**	1.000
	Subauma – Praia do Forte	**0.000**	1.000
	Siribinha – Santa Maria	1.000	**0.000**
	Subauma – Santa Maria	0.491	**0.000**
	Siribinha – Subauma	0.938	1.000
ES	Guriri – Comboios	**0.031**	0.998
	Itaunas – Comboios	0.710	1.000
	Pontal do Ipiranga – Comboios	0.111	0.112
	Povoacao – Comboios	0.990	**0.000**
	Itaunas – Guriri	0.620	1.000
	Pontal do Ipiranga – Guriri	0.958	0.225
	Povoacao – Guriri	0.056	**0.009**
	Pontal do Ipiranga – Itaunas	0.929	0.226
	Povoacao – Itaunas	0.837	**0.007**
	Povoacao – Pontal do Ipiranga	0.193	0.992
RJ	Maria Rosa – Ilha de Convivencia	**0.040**	**0.048**

Table S3. Results of General Additive Models for climate effects on hatching success (HS) and emergence rate (ER) at selected beaches. Model parameters were: air temperature (temp), average rain (avg_rain), accumulated rain (acc_rain), sea surface temperature (sst), solar radiation (rad), humidity (humid), and wind speed (wind). These predictors were explored at various temporal scales: the month nests were laid (0_climate variable), the month nests were laid and one month prior (0_1_climate variable), the month nests were laid and two months prior (0_2_climate variable), two months prior to the nests being laid (2_climate variable) and

incubation period (inc_climate variable). The models with the lowest AICc are bolded, while the model with low AICc and high significance is highlighted in gray and bolded. P – values for combined models are presented with a semicolon separating the separate p – values for each parameter in the order they are listed in. A term in the following models had fewer unique covariate combinations than specific maximum degrees of freedom and we could not run the analyses: 2_humid (Espirito Santo, Povoacao, Comboios and all combined), 0_2_humid (Espirito Santo, Itaunas, Povoacao and Comboios), 0_1_wind (Espirito Santo, Itaunas, Povoacao and Comboios) and 0_2_wind (Itaunas).

Model	Site	AICc HS	Deviance (%) HS	P – value(s) HS	AICc ER	Deviance (%) ER	P – value(s) ER
0_humid	All	2214.21	6.16	5.43 E -11	1624.96	5.24	6.83 E -8
0_temp	All	2352.83	12.30	< 2 e -16	1935.04	5.19	1.27 E -8
0_sst	All	2413.56	14.30	< 2 E -16	1817.38	24.30	< 2 E -16
0_avg_rain	All	2513.32	6.42	1.63 E -12	2000.2	6.11	8.43 E -11
0_acc_rain	All	2158.82	3.07	0.000502	1622.3	3.23	0.000222
0_rad	All	2158.85	5.96	9.39 E -10	1586.22	7.83	3.01 E -11
0_wind	All	2244.5	6.21	3.19 E -12	1669.2	7.72	7.51 E -9
0_1_humid	All	2185.65	9.12	< 2 E -16	1616.4	7.46	6.19 E -9
0_1_temp	All	2307.5	16.50	< 2 E -16	1909.06	7.91	8.68 E -14
0_1_sst	All	2401.79	15.20	< 2 E -16	1811.94	25	< 2 E -16
0_1_avg_rain	All	2512.27	6.28	2.49E-12	2019.04	4.59	1.25 E -6
0_1_acc_rain	All	2108.32	3.64	0.000315	1584.44	3.08	0.0148
0_1_rad	All	2095.97	11.40	< 2 E -16	1569.33	9.38	1.65 E -15
0_1_wind	All	2188.03	10.40	< 2 E -16	1650.66	7.25	2.19 E -8
0_2_humid	All	2227.47	5.32	3.79E-09	1610.9	8.38	3.3 E -11
0_2_temp	All	2332.43	13.70	< 2 E -16	1906.89	8.15	2.95 E -14
0_2_sst	All	2400.01	15.60	< 2 E -16	1818.05	24.10	< 2 E -16
0_2_avg_rain	All	2515.38	5.67	1.9 E -12	2031.73	3.51	0.000144
0_2_acc_rain	All	2073.97	1.28	0.034	1499.83	5.23	0.000211

0_2_rad	All	2116.76	9.37	< 2 E -16	1561.27	10.40	< 2 E -16
0_2_wind	All	2188.25	10.20	< 2 E -16	1643.01	8.51	1.78 E -9
2_humid	All	NA	NA	NA	NA	NA	NA
2_temp	All	2343.04	13	< 2 E -16	1901.31	8.88	2.44 E -15
2_sst	All	2390.16	16.20	< 2 E -16	1822.96	23.80	< 2 E -16
2_avg_rain	All	2533.77	4.14	1.29 E -8	2001.31	6.48	6.44 E -10
2_acc_rain	All	2112.9	4.71	1.03 E -6	1578.95	5.79	5.2 E -6
2_rad	All	2173.76	8.06	3.27 E -15	1632.71	12.80	< 2 E -16
2_wind	All	2216.45	9.87	< 2 E -16	1692.21	6.37	2.21 E -7
inc_humid	All	2193.83	4.35	1.08 E -6	1534.87	12.60	< 2 E -16
inc_temp	All	2377.31	4.99	1.29 E -8	1885.25	1.96	0.0359
inc_sst	All	2354.21	14.50	< 2 E -16	1755.1	23.60	< 2 E -16
inc_avg_rain	All	2443.53	5.07	1.73 E -8	1901.41	6.44	7.62 E -12
inc_acc_rain	All	1993.91	3.24	0.000309	1432.59	13.80	7.26 E -16
inc_rad	All	2094.31	4.42	1.85 E -5	1468.83	13.20	5.77 E -16
inc_wind	All	2161.88	6.60	7.59 E -12	1582.43	6.96	1.51 E -7
Inc_temp + 0_avg_rain	All	2300.12	13.30	4.63 E -6; 2 E -16	1819.25	9.11	0.0123; 5.08 E -15
Inc_temp + 0_acc_rain	All	2028.52	8.90	1.7 E -7; 2.76 E -5	1541.87	7.84	0.123; 5.47 E -5
Inc_temp + 0_1_avg_rain	All	2307.9	12.70	3.15 E -10; 9.33 E -14	1854.36	7.11	0.000403; 1.72 E -6
Inc_temp+ 0_1_acc_rain	All	1965.42	10.70	1.03 E -9; 3.95 E -8	1509.69	5.36	0.21578; 0.00834
Inc_temp + 0_2_avg_rain	All	2323.23	10.60	6.78 E -8; 1.47 E -11	1856	7.27	0.0237; 2.06 E -6
Inc_temp + 0_2_acc_rain	All	1934.64	8.51	4.3 E -8; 6.2 E -6	1416.40	9.70	0.0766; 1.13 E -6
Inc_temp + 2_avg_rain	All	2350.4	8.74	2.7 E -5; 1.68 E -6	1818.7	11.20	0.00164; 7.83 E -14
Inc_temp+ 2_acc_rain	All	1995.2	9.73	3.67 E -5; 8.38 E -7	1488.38	10.70	0.00264; 6.14 E -9
Inc_temp + inc_avg_rain	All	2321.54	9.62	2.28 E -5; 4.46 E -9	1812.1	10.40	0.00136; 2.56 E -14

Inc_temp + inc_acc_rain	All	**1915.33**	**8.94**	**2.23 E -8; 0.000899**	**1402.71**	**14.20**	**0.271; 4.48 E -16**
0_humid	Bahia	1299.08	9.46	2.85 E -11	882.06	14.50	3.14 E -10
0_temp	Bahia	1283.11	21.10	< 2 E -16	1021.74	10.30	1.84 E -13
0_sst	Bahia	1385.012	18.70	< 2 E -16	1074.99	16.70	6.94 E -16
0_avg_rain	Bahia	1460.91	10.20	2.13 E -13	1151.78	1.51	0.0841
0_acc_rain	Bahia	1270.89	1.85	0.00461	909.08	5.78	0.00988
0_rad	Bahia	1270.68	4.50	0.000201	895.65	4.53	0.00034
0_wind	Bahia	1343.82	8.54	1.32 E -9	978	3.44	0.0734
0_1_humid	Bahia	1290.55	10.20	8.71 E -13	903.51	9.19	1.03 E -5
0_1_temp	Bahia	1266.41	23.40	< 2 E -16	1008.68	15.70	2.01 E -13
0_1_sst	Bahia	1397.41	17.40	< 2 E -16	1077.96	15.10	3.4 E -15
0_1_avg_rain	Bahia	1490.02	6.46	2.11 E -7	1094.88	12.90	3.59 E -13
0_1_acc_rain	Bahia	1214.49	9.02	1.72 E -8	878.15	2.31	0.0263
0_1_rad	Bahia	1200.08	15.40	< 2 E -16	888.31	6.27	1.86 E -5
0_1_wind	Bahia	1326.11	7.49	8.48 E -9	961.7	3.35	0.13
0_2_humid	Bahia	1299.42	9.77	1.35 E -11	898.88	10.50	1.82 E -6
0_2_temp	Bahia	1291.96	20	< 2 E -16	1010.07	13.90	4.08 E -13
0_2_sst	Bahia	1419.07	14.80	< 2 E -16	1083.15	13.90	1.52 E -14
0_2_avg_rain	Bahia	1482	6.10	8.77 E -10	1129.2	7.02	4.24 E -6
0_2_acc_rain	Bahia	1171.46	10.80	2.16 E -11	813.92	9.26	0.000249
0_2_rad	Bahia	1200.09	14.30	< 2 E -16	882.63	7.45	7.79 E -7
0_2_wind	Bahia	1314.53	9.03	4.9 E -11	923.75	12	6.32 E -9
2_humid	Bahia	1343.85	6.08	9.29 E -7	934.84	10.60	1.02 E -7
2_temp	Bahia	1326.75	14.50	< 2 E -16	1004.86	14.50	4.07 E -14
2_sst	Bahia	1410.91	16	< 2 E -16	1090.05	12.60	2.31 E -13
2_avg_rain	Bahia	1502.26	3.88	2.82 E -5	1108.34	10.60	1.99 E -10
2_acc_rain	Bahia	1254.37	6.75	1.86 E -6	889.25	8.04	0.000272
2_rad	Bahia	1260.49	11.90	8.25 E -14	939.61	10.50	1.02 E -7
2_wind	Bahia	1328.22	12.80	< 2 E -16	997.21	2.31	0.00574
inc_humid	Bahia	1288.31	4.55	0.000473	816.1	20.50	3.6 E -14

inc_temp	Bahia	1312.54	9.53	4.83 E -11	944.64	16	1.81 E -12
inc_sst	Bahia	1336.55	18	< 2 E -16	1029.07	12.10	4.97 E -11
inc_avg_rain	Bahia	1414.567	5.99	9.34 E -7	1061.65	5.31	0.00452
inc_acc_rain	Bahia	1063.67	11.30	8.57 E -11	766.07	11.90	8.98 E -6
inc_rad	Bahia	1149.16	10.30	1.96 E -10	797.32	10.90	1.15 E -5
inc_wind	Bahia	1254.88	10.20	6.52 E -11	889.45	0.68	0.142
Inc_temp + 0_avg_rain	Bahia	1258.72	19.50	1.92 E -6	945.76	16.30	1.7 E -11
Inc_temp + 0_acc_rain	Bahia	1140.8	9.83	1.64 E -5	813.71	13.70	0.000449
inc.temp + 0_1_avg_rain	Bahia	1302.18	13.70	8.61 E -8	896.73	27.20	1.52 E -12
inc.temp+ 0_1_acc_rain	Bahia	1098.16	14.70	0.00158	780.71	15	0.000476
inc.temp + 0_2_avg_rain	Bahia	1286.06	14.30	1.44 E -8	915.69	24.80	1.76 E -12
inc.temp + 0_2_acc_rain	Bahia	1067.17	15	0.00422	**719.58**	**18.90**	**0.00088**
inc.temp + 2_avg_rain	Bahia	1295.67	15.10	1.86 E -10	919.65	21	4.42 E -10
inc.temp+ 2_acc_rain	Bahia	1119.8	15.20	2.11 E -5	776.64	20.60	4.75 E -6
inc.temp + inc_avg_rain	Bahia	1271.9	14.50	2.14 E -9	929.99	18.50	2.73 E -13
inc.temp + inc_acc_rain	Bahia	**943.78**	**26.50**	**4.93 E -15**	726.43	20	0.000313
0_humid	Espirito Santo	702.58	10.30	0.00196	497.41	7.19	0.0159
0_temp	Espirito Santo	758.67	15.70	5.26 E -9	567.61	9.80	0.0363
0_sst	Espirito Santo	726.61	27.70	1.09 E -15	554.16	17.60	0.000357
0_avg_rain	Espirito Santo	751.49	22.50	8.18 E -10	569.1	2.50	0.0312
0_acc_rain	Espirito Santo	693.93	9.59	0.00584	496.55	6.82	0.151
0_rad	Espirito Santo	684.83	17.60	1.2 E -6	500.54	5.35	0.0464
0_wind	Espirito Santo	685.82	17.10	1.44 E -6	487.7	19	8.92 E -5
0_1_humid	Espirito Santo	677.23	21.20	2.23 E -9	501.71	5.46	0.111
0_1_temp	Espirito Santo	741.08	24.80	6.3 E -12	568.33	2.88	0.0203

0_1_sst	Espirito Santo	728.55	27.90	4.44 E -15	564.73	4.42	0.00214
0_1_avg_rain	Espirito Santo	747.28	22.70	9.13 E -11	564.82	4.38	0.00261
0_1_acc_rain	Espirito Santo	655.03	16.30	4.16 E -6	470.76	14.90	0.000887
0_1_rad	Espirito Santo	647.42	30	3.2 E -14	503.08	4.72	0.171
0_1_wind	Espirito Santo	NA	NA	NA	NA	NA	NA
0_2_humid	Espirito Santo	NA	NA	NA	NA	NA	NA
0_2_temp	Espirito Santo	757.77	16.50	5.42 E -9	568.26	4.40	0.0694
0_2_sst	Espirito Santo	731.95	26.70	1.71 E -14	564.01	4.76	0.00138
0_2_avg_rain	Espirito Santo	747.33	19	8.37 E -12	563.42	5.04	0.00117
0_2_acc_rain	Espirito Santo	656.3	5.62	0.00239	449.66	17.90	0.000105
0_2_rad	Espirito Santo	683.3	18.10	5.49 E -7	506.51	0.16	0.593
0_2_wind	Espirito Santo	691.95	15.20	1.9 E -5	489.89	19.50	7.2 E -5
2_humid	Espirito Santo	NA	NA	NA	NA	NA	NA
2_temp	Espirito Santo	758.91	18.40	1.24 E -8	554.06	17.70	0.000117
2_sst	Espirito Santo	731.28	27.10	1.25 E -14	562.92	13.50	0.00406
2_avg_rain	Espirito Santo	730.45	27.40	3.68 E -14	563.69	7.07	0.00927
2_acc_rain	Espirito Santo	618.83	26	9.8 E -11	**445.61**	**24.10**	**4.16 E -6**
2_rad	Espirito Santo	689.92	16.40	2 E -6	506.69	0.20	0.866
2_wind	Espirito Santo	698.03	3.99	0.02	498.42	0.00	0.971
inc_humid	Espirito Santo	689.24	14.30	6.67 E -6	487.77	12.20	0.000108
inc_temp	Espirito Santo	744.78	20.70	1.31 E -11	561.75	7.03	0.00341
inc_sst	Espirito Santo	741.72	22.80	1.45 E -12	559.33	7.95	0.000403
inc_avg_rain	Espirito Santo	762.22	13.90	3.95 E -8	573.6	0.21	0.517
inc_acc_rain	Espirito Santo	682.48	14	2.46 E -5	478.17	20.20	0.000264
inc_rad	Espirito Santo	670.7	20.60	1.3 E -8	482.45	13.10	3.97 E -5

inc_wind	Espirito Santo	711.19	3.64	0.281	501.33	9.36	0.127
Inc_temp + 0_avg_rain	Espirito Santo	725.33	33.40	8.68 E -7	563.2	7.18	0.0269
Inc_temp + 0_acc_rain	Espirito Santo	654.96	24	1.61 E -10	489.49	20.50	0.00524
inc.temp + 0_1_avg_rain	Espirito Santo	717.17	40.70	5.84 E -7	563.49	6.66	0.129
inc.temp+ 0_1_acc_rain	Espirito Santo	623.36	30.90	1.42 E -7	456.05	31.60	9.04 E -5
inc.temp + 0_2_avg_rain	Espirito Santo	738.21	24.10	0.00613	561.1	12.80	0.0718
inc.temp + 0_2_acc_rain	Espirito Santo	625.47	25.60	1.66 E -7	447.9	21.70	0.151767
inc.temp + 2_avg_rain	Espirito Santo	720.09	35.30	0.00803	556.87	22.80	0.01498
inc.temp+ 2_acc_rain	Espirito Santo	**616.46**	**28.90**	**0.042235**	447.22	26	0.396218
inc.temp + inc_avg_rain	Espirito Santo	740.49	23.30	1.97 E -5	563.35	7.85	0.00288
inc.temp + inc_acc_rain	Espirito Santo	657.096	27.60	5.74 E -6	477.3	22.30	0.06309
0_humid	Mangue Seco	162.1	59.20	9.39 E -5	122.41	18.10	0.29
0_temp	Mangue Seco	165.01	61.90	5.76 E -5	124.29	32.40	0.0527
0_sst	Mangue Seco	189.32	9.34	0.00924	119.64	52.10	0.00075
0_avg_rain	Mangue Seco	185.03	38.40	0.00219	132.92	30.20	0.0739
0_acc_rain	Mangue Seco	181.83	0.04	0.868	127.28	11.20	0.45
0_rad	Mangue Seco	156.58	30.20	5.94 E -6	117.2	34.80	0.064
0_wind	Mangue Seco	188.39	38.90	0.00452	131.89	15.30	0.405
0_1_humid	Mangue Seco	166.66	41.50	0.00334	122.27	64	0.00191
0_1_temp	Mangue Seco	175.55	40.90	0.00333	122.24	58.70	0.00486
0_1_sst	Mangue Seco	185.02	34.60	0.00289	125.91	35.90	0.013
0_1_avg_rain	Mangue Seco	202.3	26.50	0.0735	136.3	28.90	0.182
0_1_acc_rain	Mangue Seco	181.31	29.20	0.0362	119.98	26.60	0.17
0_1_rad	Mangue Seco	154.72	33	2.04 E -6	115.05	28.20	0.12
0_1_wind	Mangue Seco	184.85	14.40	0.147	126.89	23.80	0.175

0_2_humid	Mangue Seco	170.45	25.70	0.0299	124.09	16.60	0.389
0_2_temp	Mangue Seco	174.99	27.30	0.036	116.9	40.90	0.00965
0_2_sst	Mangue Seco	176.43	49.40	8.77 E -5	126.19	37.50	0.0141
0_2_avg_rain	Mangue Seco	193.48	7.68	0.265	132.42	28	0.11
0_2_acc_rain	Mangue Seco	174.59	36	0.0281	117.26	0.62	0.613
0_2_rad	Mangue Seco	156.08	38.40	4.43 E -5	114.84	34.30	0.0666
0_2_wind	Mangue Seco	178.5	32.10	0.00658	127.23	34.60	0.0749
2_humid	Mangue Seco	170.37	3.69	0.523	121.95	2.99	0.772
2_temp	Mangue Seco	174.89	45.40	0.00231	123.26	50.10	0.00618
2_sst	Mangue Seco	175.08	46.30	0.000136	127.69	24.20	0.0242
2_avg_rain	Mangue Seco	187.14	40	0.0017	130.58	29.10	0.0436
2_acc_rain	Mangue Seco	169.7	43.70	0.00236	121.09	3.71	0.209
2_rad	Mangue Seco	161.83	38.10	0.000319	114.07	9.11	0.0589
2_wind	Mangue Seco	186.51	28.80	0.0123	130.53	7.33	0.295
inc_humid	Mangue Seco	149.72	37.70	0.00136	117.6	13.70	0.459
inc_temp	Mangue Seco	162.87	36.30	0.00123	119.87	45.90	0.00725
inc_sst	Mangue Seco	190.06	45.30	0.000397	125.74	34.90	0.01
inc_avg_rain	Mangue Seco	187.97	47.10	0.000382	137.88	29.80	0.153
inc_acc_rain	Mangue Seco	153.64	50.10	0.00116	114.04	2.81	0.268
inc_rad	Mangue Seco	**134.32**	**40.40**	**0.000124**	**104.13**	**30.90**	**0.109**
inc_wind	Mangue Seco	166.4	9.38	0.0268	120.24	1.30	0.42
Inc_temp + 0_avg_rain	Mangue Seco	175.07	70.30	0.1459; 0.0291	150.31	64.90	0.252; 0.346
Inc_temp + 0_acc_rain	Mangue Seco	181.22	83.10	5.42 E -6; 0.0435	114.31	54.60	0.00464; 0.04098
inc.temp + 0_1_avg_rain	Mangue Seco	206.9	84.10	0.000302 ; 0.0054	126.82	72.40	0.017; 0.0163
inc.temp+ 0_1_acc_rain	Mangue Seco	143.21	59.40	0.000152 ; 0.6875	137.03	78.70	0.119; 0.191

inc.temp + 0_2_avg_rain	Mangue Seco	162.93	50.80	0.000462 ; 0.0406	122.5	72.70	0.00801; 0.0111
inc.temp + 0_2_acc_rain	Mangue Seco	**136.31**	**55.70**	**0.000155 ; 0.3374**	560.5	97.70	0.0552; 0.1079
inc.temp + 2_avg_rain	Mangue Seco	186.2	64.80	0.0231; 0.0264	189.97	85.70	0.0249; 0.1193
inc.temp+ 2_acc_rain	Mangue Seco	157.91	73.80	0.0277; 0.0769	128.36	55.40	0.0413; 0.4003
inc.temp + inc_avg_rain	Mangue Seco	200.57	78.40	0.17132; 0.0087	139.21	70.20	0.0131; 0.4228
inc.temp + inc_acc_rain	Mangue Seco	149.73	73.70	0.00141; 0.0311	**109.46**	**51.50**	**0.0104; 0.0413**
0_humid	Praia do Forte	461.23	23.50	4.52 E -8	314.32	37.40	4.18 E -8
0_temp	Praia do Forte	472.15	38.50	< 2 E-16	374.46	21	2.34 E -5
0_sst	Praia do Forte	540.77	24.30	5.87 E - 12	390.66	28.30	9.56 E -8
0_avg_rain	Praia do Forte	525.96	32.20	2.87 E - 15	426.6	3.89	0.0649
0_acc_rain	Praia do Forte	470.34	5.78	0.0358	322.25	23.10	0.000573
0_rad	Praia do Forte	459.11	18.90	2.27 E -6	325.33	20.10	0.00212
0_wind	Praia do Forte	499.34	20.20	1.19 E -7	363.42	6.72	0.251
0_1_humid	Praia do Forte	437.29	34.20	1.68 E - 12	316.74	34.10	1.91 E -7
0_1_temp	Praia do Forte	454.81	46.90	< 2 E -16	381.99	13.70	0.000118
0_1_sst	Praia do Forte	548.43	20.50	5.61 E - 11	401.13	22.70	1.28 E -6
0_1_avg_rain	Praia do Forte	544.13	26.20	3.57 E - 11	375.24	36.70	8.94 E -14
0_1_acc_rain	Praia do Forte	442.75	20.20	1.04 E -5	334.48	3.90	0.282
0_1_rad	Praia do Forte	417.02	39.10	3.76 E - 16	321.14	14.30	0.00326
0_1_wind	Praia do Forte	481.21	20.80	9.91 E -8	350.93	18.90	0.000519
0_2_humid	Praia do Forte	447.94	27.40	1.02 E -9	326	28.30	5.25 E -6
0_2_temp	Praia do Forte	493.85	33	5.47 E - 15	378.23	18.60	5.80 E -5
0_2_sst	Praia do Forte	552.45	22.80	1.12 E - 10	405.94	20.30	9.96 E -6
0_2_avg_rain	Praia do Forte	537.16	24.50	1.08 E - 13	418.13	13.10	0.000639
0_2_acc_rain	Praia do Forte	419.41	26.10	1.16 E -7	292.32	30.30	0.000164

0_2_rad	Praia do Forte	455.45	21.80	2.57 E -7	307.56	32.50	8.74 E -6
0_2_wind	Praia do Forte	469.97	28.20	2.12 E -10	318.38	40.10	1.25 E -10
2_humid	Praia do Forte	461.64	25.80	1.52 E -8	315.82	42.30	5.63 E -11
2_temp	Praia do Forte	527.5	19.80	1.4 E -8	379.66	17.70	7.10 E -5
2_sst	Praia do Forte	544.44	25.10	5.84 E -12	406.96	19.40	1.93 E -5
2_avg_rain	Praia do Forte	554.24	18.80	1.69 E -9	391.95	29.30	1.07 E -10
2_acc_rain	Praia do Forte	434.73	29	4.08 E -9	309.81	33.10	6.52 E -6
2_rad	Praia do Forte	486.75	17.30	3.53 E -6	326.78	27.40	9.49 E -6
2_wind	Praia do Forte	476.46	27.40	2.15 E -11	350.56	25.20	3.46 E -5
inc_humid	Praia do Forte	450.36	18.40	1.66 E -5	287.81	49.20	1.02 E -11
inc_temp	Praia do Forte	514.45	11.70	2.29 E -5	348.47	26.40	8.85 E -5
inc_sst	Praia do Forte	523.99	19.90	1.71 E -9	374.72	19.70	6 E -5
inc_avg_rain	Praia do Forte	552.98	5.08	0.012	362.76	24.10	2.26 E -5
inc_acc_rain	Praia do Forte	**378.23**	**23.60**	**3.29 E -6**	279.31	30.30	4.25 E -5
inc_rad	Praia do Forte	416.03	24.70	1.85 E -7	276.22	42.70	5.62 E -9
inc_wind	Praia do Forte	454.92	22	2.25 E -7	307.85	31.30	1.21 E -6
Inc_temp + 0_avg_rain	Praia do Forte	460.79	44.90	0.0176; 2.47 E -12	357.51	36.40	5.23 E -5; 0.232
Inc_temp + 0_acc_rain	Praia do Forte	413.81	42.30	4.08 E -6; 6.54 E -7	295.42	37.20	0.05591; 0.00137
inc.temp + 0_1_avg_rain	Praia do Forte	493.9	29.80	0.084; 4.31 E -6	293.75	62.50	1.85 E -6; 1.36 E -15
inc.temp+ 0_1_acc_rain	Praia do Forte	400.77	33	0.00335; 2.46 E -5	307.5	35.40	0.0128; 0.0576
inc.temp + 0_2_avg_rain	Praia do Forte	477.1	30.40	0.0375; 4.35 E -9	328.9	50.20	3 E -6; 4.71 E -6
inc.temp + 0_2_acc_rain	Praia do Forte	395.73	35.60	0.138; 5.36 E -5	**258.95**	**60.50**	**0.000745; 2.33 E -5**
inc.temp + 2_avg_rain	Praia do Forte	477.41	33.30	4.09 E -5; 4.82 E -8	308.22	48.40	0.000213; 1.08 E -11

inc.temp+ 2_acc_rain	Praia do Forte	399.06	48.90	0.00158; 5.29 E -10	276.22	64.50	0.00013; 9.73 E -6
inc.temp + inc_avg_rain	Praia do Forte	494.83	26.80	8.06 E -5; 0.000139	339.35	40.10	0.01013; 0.00446
inc.temp + inc_acc_rain	Praia do Forte	**341.23**	**58.30**	**2.48 E -9; 1.05 E -11**	**263.46**	**31.60**	**0.22485; 0.00106**
0_humid	Santa Maria	334.3	17.40	0.00117	304.32	9.75	0.0269
0_temp	Santa Maria	377.36	12.50	0.00951	354.72	15.40	0.000769
0_sst	Santa Maria	401.06	10.40	0.0288	364.32	22.80	2.88 E -5
0_avg_rain	Santa Maria	408.62	0.14	0.686	388.38	12.30	0.117
0_acc_rain	Santa Maria	340.43	8.38	0.0351	290.03	7.16	0.119
0_rad	Santa Maria	322.63	16.20	0.0864	288.36	9.01	0.0421
0_wind	Santa Maria	356.79	10.90	0.0174	315.86	24.60	0.012
0_1_humid	Santa Maria	336.24	25.70	0.00287	301.63	10.40	0.002
0_1_temp	Santa Maria	382.4	7.47	0.0856	350.31	19.50	0.000167
0_1_sst	Santa Maria	400.12	11.10	0.0154	363.42	24.20	1.64 E -5
0_1_avg_rain	Santa Maria	394.81	20.30	0.00108	383.28	13	0.031
0_1_acc_rain	Santa Maria	320.92	38.50	7.33 E -5	281.2	8.39	0.0666
0_1_rad	Santa Maria	322.61	3.89	0.0606	287.23	8.55	0.0103
0_1_wind	Santa Maria	350.67	18.40	0.0117	314.33	4.91	0.0356
0_2_humid	Santa Maria	333.95	14	0.000163	301.81	10.20	0.00223
0_2_temp	Santa Maria	379.86	9.43	0.025	346.48	22.60	3.81 E -5
0_2_sst	Santa Maria	401.75	9.74	0.0284	367.12	21.10	9.96 E -5
0_2_avg_rain	Santa Maria	403.32	17.80	0.0197	388.74	2.31	0.335
0_2_acc_rain	Santa Maria	317.91	28.70	0.0011	259.8	17.40	0.0216
0_2_rad	Santa Maria	319.77	14.50	0.0387	278.04	42.60	0.000765

0_2_wind	Santa Maria	336.67	39	2.78 E -5	312.41	7.07	0.0116
2_humid	Santa Maria	344.76	16	0.00065	318.64	12.40	0.00429
2_temp	Santa Maria	382.22	6.64	0.0696	342.79	25.60	9.11 E -6
2_sst	Santa Maria	400.13	7.34	0.00317	375	14.90	0.00224
2_avg_rain	Santa Maria	391.9	20.60	0.000446	387.01	4.18	0.171
2_acc_rain	Santa Maria	341.48	15.40	0.00729	285.6	16.20	0.019
2_rad	Santa Maria	345.04	10.90	0.244	295.86	44	2.24 E -6
2_wind	Santa Maria	334.54	54	5.19 E -9	321.05	13.80	0.00026
inc_humid	Santa Maria	323.58	14.40	0.000258	272.71	40.40	3.34 E -6
inc_temp	Santa Maria	369.35	7.93	0.2	309.21	33	2.48 E -7
inc_sst	Santa Maria	391.03	2.49	0.267	337.25	27.70	6.63 E -5
inc_avg_rain	Santa Maria	384.16	21.70	0.00902	357.12	20.70	0.0259
inc_acc_rain	Santa Maria	**287.32**	**2.50**	**0.54**	234.31	34.40	0.00464
inc_rad	Santa Maria	291.48	32.60	0.00144	251.92	44.70	5 E -5
inc_wind	Santa Maria	320.13	28.10	0.000197	292.37	28.30	0.00665
Inc_temp + 0_avg_rain	Santa Maria	371.54	10.20	0.154; 0.466	309.51	34.60	2.27 E -8, 0.247
Inc_temp + 0_acc_rain	Santa Maria	315.93	12.60	0.312067 ; 0.000507	259.06	26.80	0.000802, 0.335465
inc.temp + 0_1_avg_rain	Santa Maria	353.46	31.20	0.029059 ; 0.000279	301.3	48	9.65 E -9; 0.00594
inc.temp+ 0_1_acc_rain	Santa Maria	301.85	41.40	0.772; 4.25 E -5	250.33	30.20	0.000291; 0.588579
inc.temp + 0_2_avg_rain	Santa Maria	360.6	38.80	0.119975 ; 0.000797	301.76	47.40	1.2 E -9; 0.00574
inc.temp + 0_2_acc_rain	Santa Maria	304.62	40.30	0.43021; 0.00249	**232.24**	**38.70**	**0.000788; 0.190178**
inc.temp + 2_avg_rain	Santa Maria	355.02	30.10	0.585245 ; 0.000186	314.48	67.40	3.27 E -7; 0.00499
inc.temp+ 2_acc_rain	Santa Maria	318.85	28.10	0.219; 0.005	251.77	41.20	0.000243; 0.1193

inc.temp + inc_avg_rain	Santa Maria	364.55	18.30	0.0413; 0.022	310.18	49.80	8.7 E -7; 0.116
inc.temp + inc_acc_rain	Santa Maria	**278.18**	**21**	**0.0788; 0.3723**	**217.39**	**43.90**	**0.084; 0.025**
0_humid	Itaunas	266.47	26.60	3.63 E -6	153.8	34.10	2.35 E -5
0_temp	Itaunas	273.87	23.40	4.75 E -6	194.9	5.45	0.432
0_sst	Itaunas	228.79	58.60	< 2 E -16	164.49	50.80	4.9 E -7
0_avg_rain	Itaunas	223.45	61.90	4.92 E -16	201.6	18.50	0.142
0_acc_rain	Itaunas	276.44	21.10	0.000165	174.34	12.10	0.218
0_rad	Itaunas	244.98	41.10	2.8 E -11	152.41	57.20	1.34 E -6
0_wind	Itaunas	236.22	47.30	3.83 E -13	143.14	57.90	1.44 E -7
0_1_humid	Itaunas	231.53	50.20	2.51 E -14	181.05	18.10	0.283
0_1_temp	Itaunas	248.75	43.50	4.83 E -11	192.7	26.70	0.00375
0_1_sst	Itaunas	243.52	47	1.39 E -12	173.37	42.50	1.16 E -5
0_1_avg_rain	Itaunas	268.77	29.70	3.35 E -7	187.11	31.90	0.00065
0_1_acc_rain	Itaunas	248.39	34.40	6.59 E -8	141.88	55.50	3.08 E -7
0_1_rad	Itaunas	**210.99**	**62.60**	**< 2 E -16**	168.7	20.90	0.0634
0_1_wind	Itaunas	NA	NA	NA	NA	NA	NA
0_2_humid	Itaunas	NA	NA	NA	NA	NA	NA
0_2_temp	Itaunas	270.57	23	1.27 E -6	187.59	32.80	0.00147
0_2_sst	Itaunas	249.84	36.20	3.18 E -11	165.55	51.10	8.23 E -8
0_2_avg_rain	Itaunas	262.1	30.20	1.59 E -8	177.12	40.40	4.39 E -6
0_2_acc_rain	Itaunas	227.67	47.20	2.78 E -10	**138.13**	**55.50**	**2.97 E -6**
0_2_rad	Itaunas	252.24	37.50	2.73 E -10	169.33	36.90	0.000697
0_2_wind	Itaunas	NA	NA	NA	NA	NA	NA
2_humid	Itaunas	273.78	19.30	0.000445	159.52	48.80	3.35 E -6
2_temp	Itaunas	247.27	43.90	2.98 E -11	194.75	18.60	0.0645
2_sst	Itaunas	227.47	53.10	2.95 E -16	177.59	41	2.45 E -6
2_avg_rain	Itaunas	244.95	49.10	2.91 E -13	168.26	47.70	1.07 E -6
2_acc_rain	Itaunas	**197.61**	**66**	**< 2 E -16**	**136.12**	**70.10**	**4.85 E -10**
2_rad	Itaunas	280.33	19.80	0.000353	152.87	53.40	3.74 E -6

2_wind	Itaunas	259.1	33.40	6.54 E -8	170.78	32.30	0.00394
inc_humid	Itaunas	243.78	28.40	8.35 E -10	151.85	32.30	1.85 E -5
inc_temp	Itaunas	261.93	32.60	2.35 E -8	181.09	31.70	0.000735
inc_sst	Itaunas	226.24	56.70	7.13 E -16	177.33	26.60	0.000603
inc_avg_rain	Itaunas	292.55	12.10	0.00898	191.27	26.60	0.00469
inc_acc_rain	Itaunas	238.05	45	3.47 E -11	158.62	47.90	1.38 E -5
inc_rad	Itaunas	233.27	47.70	5.56 E -13	142.69	63.60	2.07 E -8
inc_wind	Itaunas	233.25	49.60	2.64 E -14	157.23	48.10	8.38 E -7
Inc_temp + 0_avg_rain	Itaunas	216.478	69.30	0.00052; 1.75 E -13	213.82	55.90	0.000158; 0.030687
Inc_temp + 0_acc_rain	Itaunas	288.17	61.30	1.79 E -9, 6.43 E -5	187.32	51	0.000163; 0.336146
inc.temp + 0_1_avg_rain	Itaunas	259.13	76.70	1.33 E -10; 2.15 E -11	222.79	65.50	0.0022; 0.00203
inc.temp+ 0_1_acc_rain	Itaunas	332.94	77.80	4.68 E -10; 1.36 E -8	202.5	79	0.04134; 0.00307
inc.temp + 0_2_avg_rain	Itaunas	266.65	56.70	4.51 E -6; 8.2 E -6	183.56	68.30	0.000459; 0.000101
inc.temp + 0_2_acc_rain	Itaunas	394.64	89.50	7.41 E -10; 7.77 E -12	235.4	86.80	0.023927; 0.000712
inc.temp + 2_avg_rain	Itaunas	261.44	83.30	3.96 E -8; 1.24 E -12	177.39	75	0.0063; 1.5 E -5
inc.temp+ 2_acc_rain	Itaunas	390.68	95.70	8.98 E -7; 9.71 E -14	212.63	95.20	0.0213; 6.28 E -5
inc.temp + inc_avg_rain	Itaunas	301.08	75.20	4.75 E -15; 9.56 E -11	201.56	58.70	0.000141; 0.004796
inc.temp + inc_acc_rain	Itaunas	306.3	88.10	6.85 E -10; 1.74 E -12	210.81	72.40	0.0376; 0.0196
0_humid	Povoacao	224.71	4.49	0.383	174.67	4.03	0.623
0_temp	Povoacao	257.73	24.60	0.0311	199.97	8.18	0.023
0_sst	Povoacao	247.22	40.50	0.000405	195.42	15.40	0.00172
0_avg_rain	Povoacao	260.96	5.02	0.327	195.19	15.70	0.00192

0_acc_rain	Povoacao	218.5	17.30	0.197	164.33	43	0.0181
0_rad	Povoacao	229.2	18.40	0.353	176.75	16.50	0.573
0_wind	Povoacao	219.84	21	0.0502	172.9	12.90	0.198
0_1_humid	Povoacao	223.33	4.32	0.108	172.94	10.60	0.381
0_1_temp	Povoacao	251.49	29	0.00309	198.04	12.60	0.011
0_1_sst	Povoacao	244.11	48.10	4.5 E -5	197.4	12.20	0.00494
0_1_avg_rain	Povoacao	251.25	27.20	0.00667	196.26	15.80	0.0192
0_1_acc_rain	Povoacao	209.51	10.80	0.216	161.66	12.40	0.125
0_1_rad	Povoacao	215.03	29.80	0.0102	171.8	18.40	0.139
0_1_wind	Povoacao	NA	NA	NA	NA	NA	NA
0_2_humid	Povoacao	NA	NA	NA	NA	NA	NA
0_2_temp	Povoacao	255.54	13.20	0.0212	197.94	21.10	0.0309
0_2_sst	Povoacao	245.83	31.60	0.000729	199.31	9.21	0.0145
0_2_avg_rain	Povoacao	247.39	25.20	0.00069	194.11	18.60	0.00621
0_2_acc_rain	Povoacao	207.19	0.59	0.568	**155.96**	**13.30**	**0.0324**
0_2_rad	Povoacao	220.76	33	0.0335	173.014	4.36	0.15
0_2_wind	Povoacao	221.08	8.07	0.0336	173.6	3.72	0.342
2_humid	Povoacao	NA	NA	NA	NA	NA	NA
2_temp	Povoacao	255.07	14.50	0.017	195.67	18.90	0.014
2_sst	Povoacao	247.43	33.80	7 E -4	200.12	7.94	0.0226
2_avg_rain	Povoacao	241.64	34.50	4.26 E -5	196.36	16.80	0.0116
2_acc_rain	Povoacao	**203.36**	**12.80**	**0.0813**	**155.6**	**22**	**0.0785**
2_rad	Povoacao	225.42	0.85	0.475	173.84	2.60	0.267
2_wind	Povoacao	215.51	8.31	0.0326	169.55	0.81	0.885
inc_humid	Povoacao	216.55	40.80	0.00815	171.31	8	0.0518
inc_temp	Povoacao	259.06	30.70	0.0078	195.51	15.20	0.00199
inc_sst	Povoacao	259.49	6.49	0.0914	196.77	16.60	0.00899
inc_avg_rain	Povoacao	251.93	22	0.00481	206.43	11.10	0.448
inc_acc_rain	Povoacao	215.96	23.70	0.088	169.08	0.98	0.511
inc_rad	Povoacao	223.64	7.81	0.314	165.62	20.20	0.00228
inc_wind	Povoacao	222.56	33	0.029	172.74	4.96	0.138
Inc_temp + 0_avg_rain	Povoacao	258.59	36.10	0.00597; 0.14591	195.01	19.70	0.1101; 0.0928
Inc_temp + 0_acc_rain	Povoacao	220.2	24.70	0.202; 0.21	165.37	48.60	0.1183; 0.0528

inc.temp + 0_1_avg_rain	Povoacao	264.44	50.70	0.284; 0.065	198.62	21.80	0.455; 0.59
inc.temp+ 0_1_acc_rain	Povoacao	210.39	22	0.1811; 0.0216	161.53	14.50	0.115; 0.299
inc.temp + 0_2_avg_rain	Povoacao	249.77	25.50	0.68192; 0.00774	196.19	18.90	0.583; 0.196
inc.temp + 0_2_acc_rain	Povoacao	216.99	24.40	0.189; 0.748	157.18	16.10	0.257; 0.296
inc.temp + 2_avg_rain	Povoacao	243.81	34.80	0.480745 ; 0.000424	197.45	18	0.218; 0.519
inc.temp+ 2_acc_rain	Povoacao	**205.54**	**13.70**	**0.5487; 0.0883**	156.71	27.10	0.185; 0.352
inc.temp + inc_avg_rain	Povoacao	251.93	27.30	0.0926; 0.0142	197.84	15.20	0.00242; 0.94441
inc.temp + inc_acc_rain	Povoacao	218.32	47.70	0.0766; 0.0145	166.25	23	0.043; 0.364
0_humid	Comboios	213	18.60	0.243	159.84	0.06	0.881
0_temp	Comboios	230.63	30.40	0.00184	177.22	1.08	0.499
0_sst	Comboios	226.50	37.40	0.000167	175.51	6.70	0.282
0_avg_rain	Comboios	243.77	0.81	0.511	177.19	1.16	0.485
0_acc_rain	Comboios	210.25	24.50	0.235	155.81	1.58	0.421
0_rad	Comboios	203.86	21	0.00182	159.36	1.25	0.478
0_wind	Comboios	215.51	12.60	0.611	158.95	5.02	0.344
0_1_humid	Comboios	205.75	33.20	0.0216	159.16	15	0.425
0_1_temp	Comboios	230.72	29.80	0.00148	177.32	0.86	0.547
0_1_sst	Comboios	227.02	35.30	0.000146	175.96	4.06	0.188
0_1_avg_rain	Comboios	233.65	36.10	0.00785	176.92	1.80	0.384
0_1_acc_rain	Comboios	199.22	21.20	0.143	149.63	8.46	0.0623
0_1_rad	Comboios	200.99	32.30	0.00156	160.53	19.10	0.355
0_1_wind	Comboios	NA	NA	NA	NA	NA	NA
0_2_humid	Comboios	NA	NA	NA	NA	NA	NA
0_2_temp	Comboios	233.61	28.70	0.00852	177.62	0.15	0.8
0_2_sst	Comboios	228.31	29.80	6.01 E -5	176.38	3.06	0.251
0_2_avg_rain	Comboios	234.33	24.80	0.00796	176.42	2.97	0.263
0_2_acc_rain	Comboios	192.52	23.20	0.0826	**141.89**	**19.20**	**0.00506**
0_2_rad	Comboios	201.44	26.30	0.000463	159.81	0.12	0.825
0_2_wind	Comboios	212.98	1.12	0.478	160.75	8.83	0.509
2_humid	Comboios	NA	NA	NA	NA	NA	NA
2_temp	Comboios	235.5	28.10	0.0182	177.74	5.24	0.834

2_sst	Comboios	227.76	30.90	4.21 E -5	175.2	5.85	0.11
2_avg_rain	Comboios	234.01	33.30	0.00596	176.25	3.38	0.23
2_acc_rain	Comboios	190.18	29.40	0.0235	142.1	18.70	0.00484
2_rad	Comboios	204.49	19.60	0.00248	159.84	0.06	0.88
2_wind	Comboios	207.59	3.55	0.462	156	3.81	0.516
inc_humid	Comboios	211.48	13.10	0.191	157.85	9.89	0.28
inc_temp	Comboios	224.73	42.30	0.000121	172.73	17	0.073
inc_sst	Comboios	227.53	35.70	0.000244	174.08	11.30	0.155
inc_avg_rain	Comboios	229.21	28.10	0.000112	176.85	1.97	0.361
inc_acc_rain	Comboios	204.26	9.78	0.0353	156.15	14.90	0.373
inc_rad	Comboios	194.35	31.40	0.000159	149.82	21.30	0.0241
inc_wind	Comboios	203.11	14.30	0.0678	154.27	40.90	0.0602
Inc_temp + 0_avg_rain	Comboios	227.07	42.30	0.000135 ; 0.594719	174.74	18.50	0.0675; 0.6219
Inc_temp + 0_acc_rain	Comboios	191.43	48.30	7.8 E -5; 0.627	151.12	26.40	0.0307; 0.2737
inc.temp + 0_1_avg_rain	Comboios	227.15	42.40	0.00153; 0.80246	175.14	17	0.0896; 0.8061
inc.temp+ 0_1_acc_rain	Comboios	185.21	50.30	6.26 E -5; 0.226	147.3	27.20	0.0591; 0.2128
inc.temp + 0_2_avg_rain	Comboios	227.11	42.30	0.00617; 0.88256	175.12	17.10	0.111, 0.866
inc.temp + 0_2_acc_rain	Comboios	**180.08**	**58.90**	**0.000324 ; 0.426618**	**141.59**	**32.90**	**0.2423; 0.0497**
inc.temp + 2_avg_rain	Comboios	229.2	57	0.0175; 0.4125	175.26	27.60	0.0791; 0.4968
inc.temp+ 2_acc_rain	Comboios	**180.66**	**52.80**	**0.00128; 0.40691**	143.15	28.30	0.429; 0.107
inc.temp + inc_avg_rain	Comboios	223.77	47	0.0179; 0.0315	174.93	17.80	0.113; 0.816
inc.temp + inc_acc_rain	Comboios	191.19	49.10	0.000531 ; 0.5156	149.54	29.70	0.00673; 0.08053
0_humid	Maria Rosa	170.82	40.10	0.0681	135.9	23.90	0.0848
0_temp	Maria Rosa	211.14	20.90	0.066	167.52	13.40	0.0197
0_sst	Maria Rosa	210.19	14	0.0607	153.39	33.90	2.73 E -6
0_avg_rain	Maria Rosa	208.08	38.40	0.00745	157.46	27.70	3.42 E -5
0_acc_rain	Maria Rosa	166.43	43.40	0.0193	139.55	43.40	0.0261

0_rad	Maria Rosa	157.6	16.30	0.00433	136.15	8.26	0.204
0_wind	Maria Rosa	165.95	3.05E-07	0.999	135.71	19.50	0.135
0_1_humid	Maria Rosa	152.42	47.80	0.000567	134.35	8.02	0.0456
0_1_temp	Maria Rosa	209.15	44.40	0.00096	158.84	29.70	0.000765
0_1_sst	Maria Rosa	209.63	16.90	0.0508	152.57	35.20	1.56 E -6
0_1_avg_rain	Maria Rosa	214.15	1.40	0.331	151.1	44.30	1.07 E -5
0_1_acc_rain	Maria Rosa	169.88	25.70	0.197	131.84	17.90	0.0474
0_1_rad	Maria Rosa	156.62	18.20	0.00241	134.25	8.23	0.0424
0_1_wind	Maria Rosa	164.54	59.50	0.000447	136.51	62.20	0.000836
0_2_humid	Maria Rosa	154.04	47.20	0.00151	129.56	17.60	0.00323
0_2_temp	Maria Rosa	208.5	9.79	0.0109	161.51	46.60	0.000845
0_2_sst	Maria Rosa	211.46	27.30	0.0324	156.5	30.70	7.09 E -6
0_2_avg_rain	Maria Rosa	210.29	9.59	0.066	152.48	42.50	1.54 E -5
0_2_acc_rain	Maria Rosa	161.95	13.70	0.0772	125.16	30.10	0.00119
0_2_rad	Maria Rosa	154.57	22.20	0.000815	132.77	49.50	0.00556
0_2_wind	Maria Rosa	165.29	1.28	0.417	131.68	45.20	0.00635
2_humid	Maria Rosa	152.45	49.90	0.000533	126.94	47.20	0.00161
2_temp	Maria Rosa	218.5	11.70	0.539	160.41	54.40	0.000137
2_sst	Maria Rosa	211.73	28	0.0189	161.51	50.80	0.000145
2_avg_rain	Maria Rosa	201.26	38.60	0.000688	160.72	22.60	0.000131
2_acc_rain	Maria Rosa	162.08	14.70	0.101	**122**	**60.70**	**0.000129**
2_rad	Maria Rosa	157.22	17	0.00332	142.86	45.60	0.017
2_wind	Maria Rosa	169.32	17.10	0.399	134.97	6.78	0.0637
inc_humid	Maria Rosa	168.89	27.20	0.17	134.6	7.52	0.0555
inc_temp	Maria Rosa	199.26	34.60	0.000511	159.26	24.90	6.60 E -5

inc_sst	Maria Rosa	202.06	28.40	0.00238	155.02	39.20	7.74 E -5
inc_avg_rain	Maria Rosa	211.77	39.50	0.0053	173.1	6.42	0.197
inc_acc_rain	Maria Rosa	152.44	37.70	0.00108	134.86	7	0.0656
inc_rad	Maria Rosa	158.62	70.20	0.000149	130.81	15.10	0.00681
inc_wind	Maria Rosa	162.87	5.99	0.0785	137.64	7.63	0.34
Inc_temp + 0_avg_rain	Maria Rosa	207.98	60	0.0075; 0.1053	156.1	33.50	0.0491; 0.019
Inc_temp + 0_acc_rain	Maria Rosa	147.76	55.50	6.65 E -5; 0.192	135.01	66.10	0.00171; 0.03911
inc.temp + 0_1_avg_rain	Maria Rosa	199.88	38.10	0.000261; 0.100189	153.87	44.30	0.858; 0.0205
inc.temp+ 0_1_acc_rain	Maria Rosa	**143.29**	**64.50**	**7.81 E -6; 0.0129**	126.53	29.40	0.00229; 0.09856
inc.temp + 0_2_avg_rain	Maria Rosa	202.06	34.70	0.00397; 0.85387	153.82	44.70	0.2263; 0.0118
inc.temp + 0_2_acc_rain	Maria Rosa	143.88	63	8.99 E -6; 0.0166	**120.31**	**41.30**	**0.0049; 0.00224**
inc.temp + 2_avg_rain	Maria Rosa	204.09	59.50	0.037; 0.341	158.78	29.40	0.0393; 0.0819
inc.temp+ 2_acc_rain	Maria Rosa	**138.98**	**70.70**	**6.09 E -7; 0.000757**	133.67	69.50	0.5226; 0.0114
inc.temp + inc_avg_rain	Maria Rosa	200.7	36.30	0.000375; 0.245785	160.69	33.20	0.000443; 0.0844
inc.temp + inc_acc_rain	Maria Rosa	145.4	60.60	0.00894; 0.04608	128.3	28	0.00238; 0.52042

Table S4. Distances (km) of weather stations from nesting beaches considered in this study.

State	Weather Station Location	Nesting Beach	Distance Between Weather Station and Nesting Beach (km)
BA	Salvador, Bahia	Mangue Seco	208
		Dunas	193
		Siribinha	138
		Baixios	121
		Subauma	106
		Costa do Sauipe	87

		Praia do Forte	76
		Itacimirim	55
		Berta	48
		Santa Maria	34
ES	Vitória, Espírito Santo	Itaunas	215
		Guriri	169
		Pontal do Ipiranga	130
		Povoacao	109
		Comboios	80
RJ	São Tomé, Rio de Janeiro	Ilha de Convivencia	25
		Maria Rosa	12

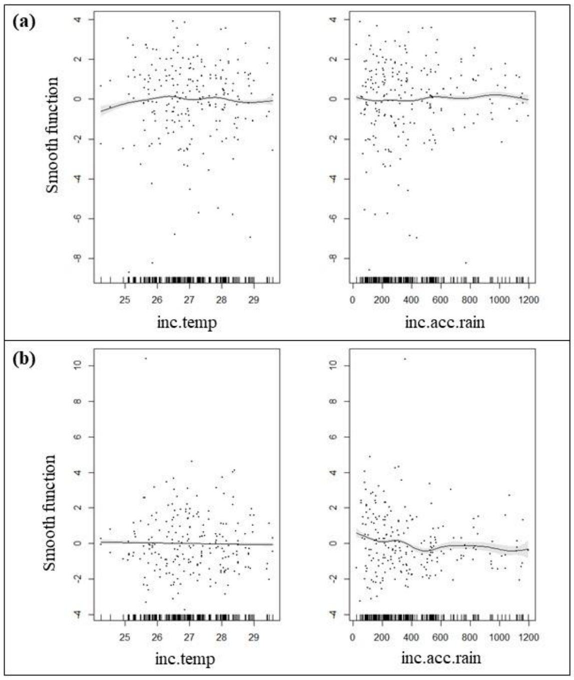

Figure S1. Best fit and most significant model for (a) hatching success and (b) emergence rate across Brazil using AICc, deviance, and p-values. The best model describing (a) hatching success was air temperature during incubation (inc.temp) in combination with accumulated

rainfall during incubation (inc.acc.rain). This same model was the best fit model describing (b) emergence rate.
